# Nutritional Programming of Lifespan by FOXO Inhibition on Sugar-Rich Diets

**DOI:** 10.1016/j.celrep.2016.12.029

**Published:** 2017-01-10

**Authors:** Adam J. Dobson, Marina Ezcurra, Charlotte E. Flanagan, Adam C. Summerfield, Matthew D.W. Piper, David Gems, Nazif Alic

**Affiliations:** 1 Institute of Healthy Ageing and Department of Genetics, Evolution and Environment, University College London, Gower Street, WC1E 6BT London, UK; 2 School of Biological Sciences, Monash University, Melbourne, VIC 3800, Australia

**Keywords:** aging, forkhead box O, nutritional programming of lifespan, *Drosophila*, diet, transcriptional regulation

## Abstract

Consumption of unhealthy diets is exacerbating the burden of age-related ill health in aging populations. Such diets can program mammalian physiology to cause long-term, detrimental effects. Here, we show that, in *Drosophila melanogaster*, an unhealthy, high-sugar diet in early adulthood programs lifespan to curtail later-life survival despite subsequent dietary improvement. Excess dietary sugar promotes insulin-like signaling, inhibits dFOXO—the *Drosophila* homolog of forkhead box O (FOXO) transcription factors—and represses expression of dFOXO target genes encoding epigenetic regulators. Crucially, *dfoxo* is required both for transcriptional changes that mark the fly’s dietary history and for nutritional programming of lifespan by excess dietary sugar, and this mechanism is conserved in *Caenorhabditis elegans*. Our study implicates FOXO factors, the evolutionarily conserved determinants of animal longevity, in the mechanisms of nutritional programming of animal lifespan.

## Highlights

• A high-sugar diet in early life programs *Drosophila* lifespan
• High sugar inactivates dFOXO, altering mRNA levels of chromatin modifiers
• *dfoxo* is required for long-term transcriptional changes in response to high sugar
• *dfoxo* and *daf-16* are required for programming of lifespan by high-sugar diets

## Graphical Abstract

**Figure:**
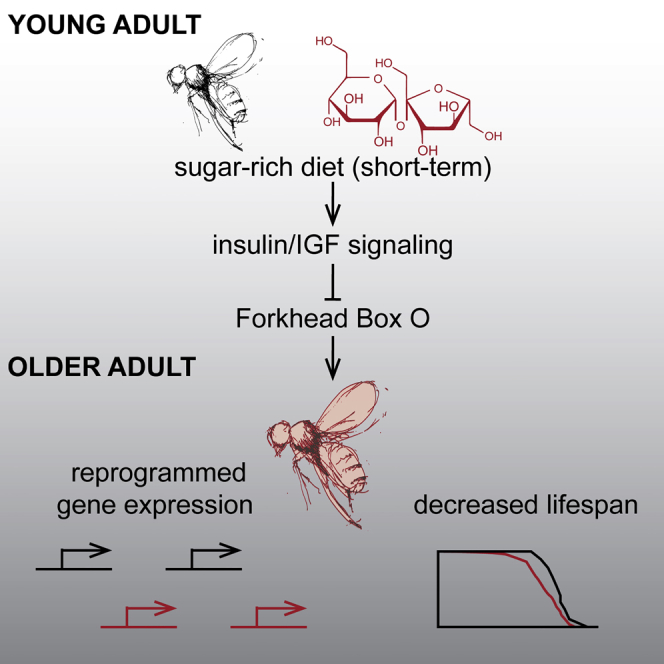

## Teaser

Modern diets can have negative consequences for long-term health. Dobson et al. show that high-sugar diets program fly and worm lifespan through the regulation of forkhead box O transcription factors.

## Introduction

Age is the main risk factor for a plethora of chronic human illnesses (Niccoli and Partridge, 2012). Aging is influenced by many parameters throughout the life of an individual, with most variation in human lifespan attributable to environmental differences (Slagboom et al., 2011). Currently, one of the main environmental insults on human health is the food we eat (Lustig et al., 2012). Indeed, chronic diseases are on the rise globally, due in part to aging populations (Christensen et al., 2009) but also due to the increasing consumption of unhealthy diets dominated by highly processed, low-cost foods (Dearden and Ozanne, 2015, Lustig et al., 2012). For example, sugar consumption has tripled over the last 50 years and is linked to a range of detrimental health outcomes (Lustig et al., 2012). These conditions underlie a pandemic of metabolic disorders, such as obesity and diabetes, which amplify the disease burden of an increasingly aged population.

An individual’s long-term adult health is influenced not only by their current diet but also by dietary history. Persistent effects of nutrition are termed nutritional programming, where a nutritional stimulus triggers a structural change or a persistent physiological state with long-term functional consequences (Lucas, 1998). In humans and other mammals, there is a wealth of epidemiological and experimental evidence that both under- and over-nutrition in early life can profoundly influence later-life health and survival. Long-term effects arise during development, potentially in early adulthood, and can be transmitted from parent to offspring (Dearden and Ozanne, 2015, Fernandez-Twinn et al., 2014, Gillman, 2005, Hardikar et al., 2015, Hirko et al., 2015, Langley-Evans, 2006). Importantly, even small, persistent effects can have important societal consequences due to the sheer number of people consuming unhealthy diets (Lustig et al., 2012). The molecular mechanisms that link past nutritional experience to curtailed survival and detrimental health outcomes remain unclear.

*Drosophila melanogaster* is an important model in understanding the interaction between nutrition and aging (Piper et al., 2011, Simpson et al., 2015, Tatar et al., 2014). To be consistent with their ecology, laboratory fruit flies are often fed a diet composed of yeast (a protein source) and sugar (Bass et al., 2007). High-protein diets shorten *Drosophila* lifespan (Lee et al., 2008, Partridge et al., 1987, Skorupa et al., 2008), but the effects of protein excess on mortality appear completely and rapidly reversible in adult *Drosophila* (Mair et al., 2003), indicating that protein-skewed diets do not program subsequent mortality (Mair et al., 2003). However, some diet-induced physiological changes are irreversible in the adult fly (van den Heuvel et al., 2014). In fruit flies, increased mortality due to excessive dietary sugar continues after dietary change (Mair et al., 2005), whereas parental sugar consumption enhances obese-like phenotypes in the offspring (Buescher et al., 2013, Öst et al., 2014). These studies hint that sugar-rich diets can program *Drosophila* physiology and should be explored as a model of the mechanisms connecting dietary history to aging.

Here, we show that consuming a diet high in sugar (sucrose) in early adulthood curtails later-life survival in *Drosophila* through nutritional programming. We show that sugar regulates the activity of the *Drosophila* forkhead box O (FOXO) transcription factor (TF) to set up gene expression changes that mark the fly’s nutritional history. We find that *dfoxo* is required to establish long-term, detrimental effects of past excessive sugar consumption. Importantly, this role of *dfoxo* is conserved in its *Caenorhabditis elegans* ortholog, *daf-16*. Our findings reveal FOXO factors as a mechanistic link between dietary history and later-life survival.

## Results

### Excessive Sugar in Early Adult Fly Diet Curtails Survival in Middle and Old Age

**Figure 1:**
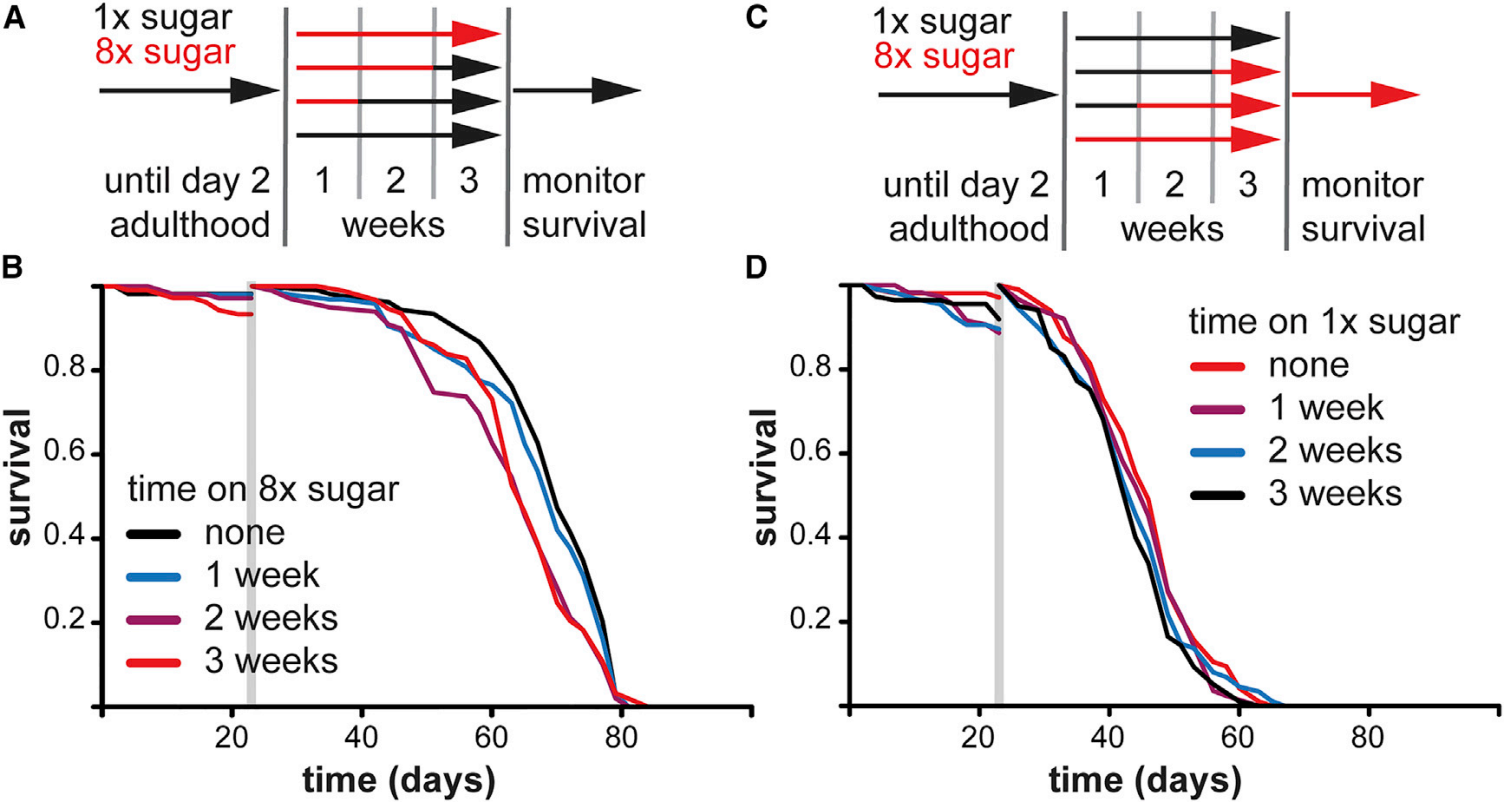
Excess Sugar in Early Adulthood Curtails Later-Life Survival (A) Experimental design. (B) Survival of females after feeding on 8×S for 1–3 weeks, compared to those continuously kept on 1×S. (C) Experimental design of the reverse switch. (D) Survival of females on 8×S after feeding on 1×S for 1–3 weeks compared to those continuously kept on 8×S. Total dead = 403; censored = 46. Only 3 weeks on 1×S showed a significant difference to control (reduced survival; p = 0.02; log rank test). In both (B) and (D), the gray vertical bar indicates the time of the last switch (23 days), when survival was reset to 1. See also Figure S1 and Table S1.

Can excess sugar in the adult diet program *Drosophila* survival? We compared lifespans of wild-type, outbred female flies that were continuously fed a diet containing sucrose concentration optimal for lifespan (5% sucrose, referred to as 1× sugar [1×S]; Bass et al., 2007) to that of their sisters, which were transiently fed an 8× excess of sugar (8×S) starting from day 2 of adulthood (Figure 1A). 8×S diet has both an increased caloric value and skewed protein-to-carbohydrate ratio. We limited treatment time to 3 weeks (a third of median life expectancy) to circumvent premature mortality that results from consuming this diet long term (Al Saud et al., 2015, Skorupa et al., 2008), thus avoiding potential bias arising from selection of hardy individuals. Fewer than 10% of experimental flies died during treatment (Figure 1B).

To evaluate the persistent, long-lasting effects of 8×S diet, we examined survival when all the flies were back on 1×S food. We found that the median lifespan of flies that had been fed 8×S for 3 weeks was reduced (7%; Figure 1B). The effect could not be attributed to changes in feeding after the exposure to 8×S food, because no differences in feeding or body mass were observed after 1 week of recovery on 1×S (Figures S1A and S1B). Interestingly, excess sugar did not impact survival immediately after treatment but created a vulnerability to the effects of age (Figure 1B). Statistical modeling using Cox proportional hazards (CPHs) confirmed that the time spent on 8×S before 23 days of age significantly increased the risk of death after 23 days (p < 2 × 10^−16^; Table S1). We noticed that different treatment groups had different median but similar maximum lifespans, prompting us to examine whether the effect of 8×S decayed with time. The increase in risk of death decayed with time (p < 2 × 10^−16^; Table S1), implying either that the flies had a heterogeneous response to sugar, potentially due to the genetic variation in the outbred population, or that the effect of 8×S feeding was slowly erased.

To ensure that the effect of 8×S feeding was substantially long term and to better estimate its magnitude, we examined the demography of survival in middle and old age, between 40 and 80 days, by analyzing over 1,000 deaths. Having been exposed to 8×S in early adulthood significantly increased relative risk of death in both mid-life (40–60 days interval) and late life (61–80 days interval), with 3 weeks on 8×S increasing the relative risk by ∼50% (Figure S1C). Notably, the magnitude of this effect was comparable to the reported 91% increase in the relative, all-cause mortality risk in middle-aged and older humans who were obese as young adults independently of their BMI later in life (Hirko et al., 2015). Hence, excess sugar consumption in early adulthood has long-term detrimental effects in the fruit fly.

Such detrimental effects could be due to either programming of fly physiology or accumulation of irreparable molecular damage. We reasoned that, if damage caused the long-term effects of sugar-rich diets in the first third of life, then feeding on a healthy diet (1×S) in the same period, before any major mortality occurs, should be beneficial regardless of the subsequent diet (Figure 1C). We found no evidence of improved survival after feeding on 1×S (Figures 1D, S1D, and S1E). This indicates that, rather than cause irreparable damage, the relative amount of sugar consumed in a fly’s early adulthood triggers a lasting physiological change or program, which can be detrimental in later life. In mammals, such persistent effects of nutrition are referred to as nutritional programming (Lucas, 1998). Overall, our data are consistent with nutritional programming of lifespan by relative sugar levels encountered in early adulthood in *Drosophila*.

### Transcriptional Response to Sugar Implicates dFOXO

**Figure 2:**
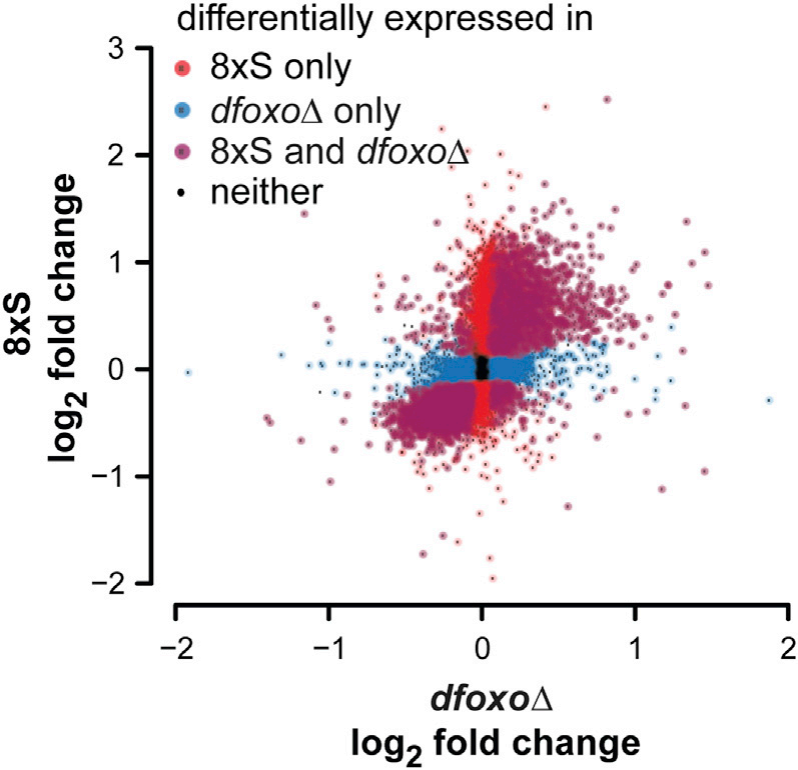
Transcriptional Response to Sugar Implicates dFOXO The transcriptional response to 8×S is plotted against the response induced by deletion of *dfoxo*. Genes with significant differential expression (FDR = 10%) in either or both conditions are indicated. Note that only the genes present in both datasets are included. See also Data S1.

Next, we sought a regulatory mechanism whereby the 8×S diet programs lifespan. A proposed mechanism for mediating nutritional programming is the regulation of gene expression (Barnes and Ozanne, 2011, Niculescu and Lupu, 2011). We chose to identify TFs responsive to 8×S as candidates for mediating the long-term effects of this diet. To explore the transcriptional signature of sugar in *Drosophila*, we used RNA sequencing (RNA-seq) to interrogate whole-body transcriptomes of females fed 8×S or 1×S diet for 1 week, because any long-lasting programs must be a consequence of the changes occurring during exposure to the diet. A total of 6,435 genes were differentially expressed on 8×S (10% false discovery rate [FDR]; Figure 2; all gene lists are given in Data S1). Interestingly, we found that the promoters of genes repressed by 8×S were enriched for forkhead-like binding motifs (Data S1). FOXO TFs are evolutionarily conserved longevity determinants: activation of FOXO orthologs can extend lifespan in budding yeast, worms, and flies, and human *Foxo3* is one of only two genes consistently associated with longevity (Giannakou et al., 2004, Hwangbo et al., 2004, Kenyon et al., 1993, Morris et al., 2015, Postnikoff et al., 2012).

To explore whether the sole fly FOXO ortholog, *dfoxo*, may be involved in the transcriptional response to 8×S, we compared the list of sugar-responsive genes to the previously published set of genes differentially expressed in *dfoxoΔ* females (Alic et al., 2011). We found that the expression of 60% of sugar-responsive genes was also altered in *dfoxoΔ* flies, representing a highly significant overlap (p = 9.4 × 10^−181^; Figure 2). The transcriptional changes tended to be in the same direction between the two treatments (Figure 2), suggesting that dFOXO is inhibited by high sugar. We used Gene Ontology (GO) enrichment analysis on the genes regulated by both 8×S and *dfoxo* to predict the functional consequences of this inhibition. We found an enrichment of genes encoding chromatin modifiers, such as a range of chromatin/nucleosome remodelers and histone-modifying enzymes (Data S1), implicating epigenetic mechanisms in the long-term effects of sugar downstream of *dfoxo*. Overall, the transcriptional response pointed toward dFOXO as a candidate mechanistic mediator of nutritional programming by a sugar-rich diet in *Drosophila*, which we pursued further.

### dFOXO Is Inhibited on Sugar-Rich Diet and Required for Transcriptional Changes that Mark Dietary History

**Figure 3:**
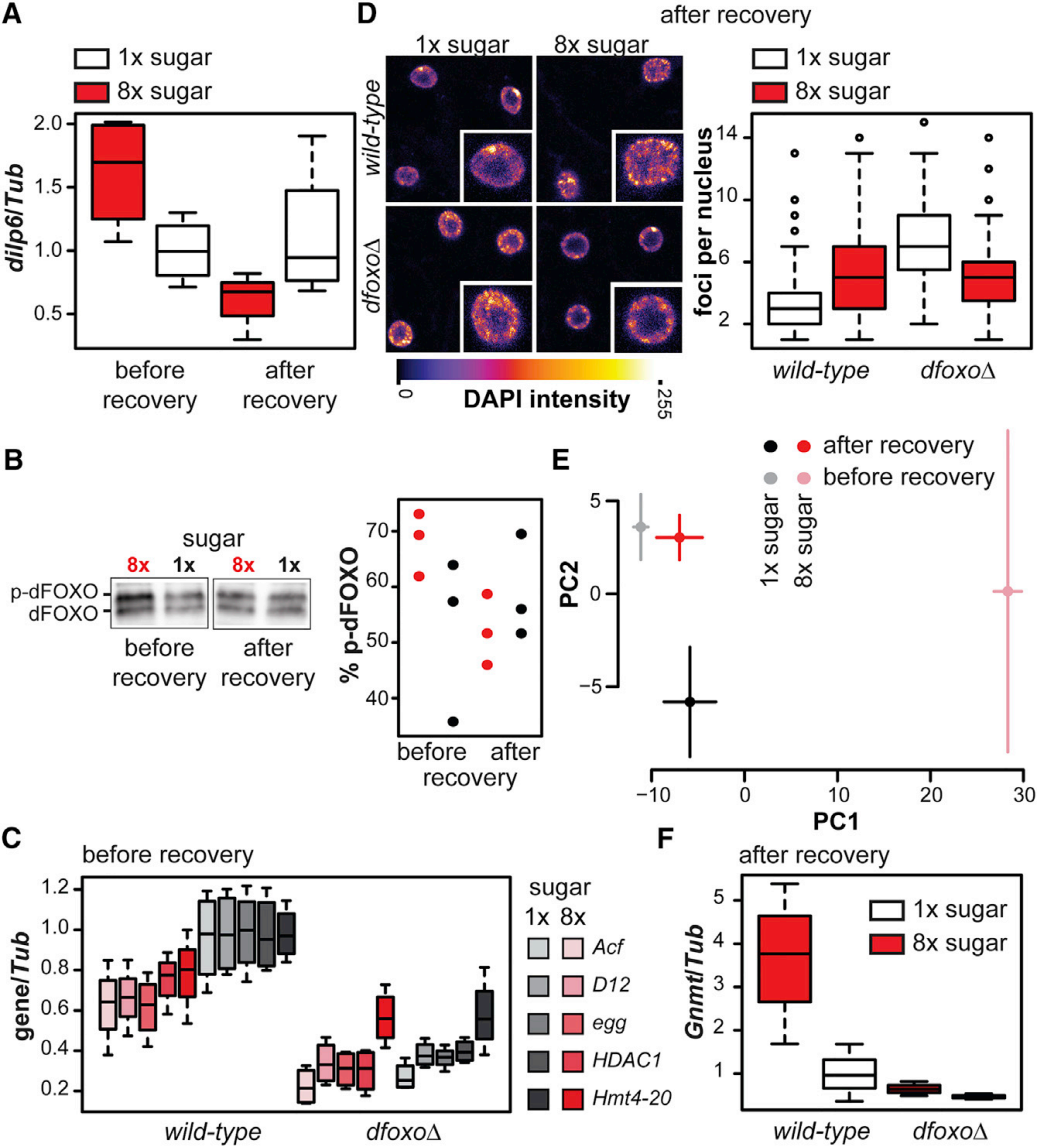
dFOXO Activity Is Regulated in Response to a Sugar-Rich Diet and Is Required for Transcriptional Memory of Past Diet (A) *dilp6* transcript levels in wild-type females kept on 8×S or 1×S for 1 week (before recovery) and then allowed to recover on 1×S for 1 week (after recovery). Data were scaled to 1×S before recovery and analyzed with a linear model: the interaction between diet and time was significant (p = 0.01; n = 4). *dilp6* was induced significantly before (p < 0.05; two-tailed t test), but not after, recovery. (B) Phosphorylated and unphosphorylated forms of dFOXO were separated by SDS-PAGE in whole-fly protein extracts obtained from females kept on 8×S or 1×S for 3 weeks and recovered on 1×S for 1 week. Quantifications from three repeats are shown to the right. The data were analyzed with a mixed-effects linear model, with repeat as a random effect: there was no significant effect of diet or time, but their interaction was significant (p = 0.024). 8×S was significantly different from 1×S before (t test; p < 0.05), but not after, recovery. (C) Transcript levels of five genes encoding chromatin modifiers in wild-type and *dfoxoΔ* females after 1 week feeding on 1×S or 8×S. Data were scaled to wild-type levels on 1×S and analyzed with a linear model: the effects of transcript, diet, and genotype were significant (p < 0.005; n = 4). Interactions between transcript and other covariates were not significant, and genotype interacted with diet (p = 6 × 10^−4^): diet had a significant effect in the wild-type (p < 0.05; t test), but not *dfoxoΔ*. (D) Distribution of DAPI staining in nuclei of abdominal fat body cells after recovery from 8×S in wild-type and *dfoxoΔ* females. DAPI intensity is false-colored for clarity. The quantification of the number of foci per nucleus is given along the representative images. Single cell inserts are ∼10 × 10 μm. Generalized linear model with a Poisson distribution revealed a significant effect of genotype and significant interaction of genotype with diet (n = 50–90 nuclei from four or five animals; p < 10^−4^). (E) PCA analysis of transcriptomes of flies exposed to 8×S or 1×S for 1 week (before recovery; see Figure 2) or those allowed to recover for 1 week on 1×S (after recovery). Points show means ± SEs. PC1 and 2 collectively account for ∼60% of total variance in the dataset. (F) *Gnmt* transcript levels in wild-type or *dfoxoΔ* females kept on 8×S or 1×S for 1 week and then allowed to recover on 1×S for 1 week. Data are scaled to wild-type on 1×S and analyzed with a liner model: interaction between genotype and diet was significant (p = 0.03; n = 3 or 4), with *Gnmt* induced in the wild-type after 8×S feeding (p < 0.05; t test), but not in *dfoxoΔ*. See also Figure S2.

We next examined whether dFOXO is regulated on 8×S diet. dFOXO is inhibited by the signaling cascade initiated by *Drosophila* insulin-like peptides (DILPs) (Teleman, 2009). We found that one of these, *dilp6*, was induced after 1 week of feeding on the 8×S diet (p < 0.05; Figure 3A). Such an increase in insulin/insulin-like growth factor (IGF) signaling is expected to result in phosphorylation and inhibition of dFOXO (Brunet et al., 1999, Alic et al., 2011). Indeed, we found that dFOXO phosphorylation was increased on the 8×S diet (p < 0.05; Figure 3B). To confirm that this phosphorylation impacts dFOXO’s transcriptional activity, we selected several genes identified as responsive to both the 8×S diet and *dfoxo* deletion and examined whether their transcript levels were modulated by 8×S in a *dfoxo*-dependent manner. We focused on genes encoding epigenetic regulators due to their likely relevance to the legacy of the 8×S diet. The transcript levels of *Acf*, encoding a chromatin assembly factor subunit, *D12*, a subunit of the ATAC histone acetyltransferase complex, *egg*, a histone methyltransferase, *HDAC1*, a histone deacetylase, and *Hmt4-20*, a histone methyltransferase, were all reduced in *dfoxoΔ* flies (p < 0.005) and in the wild-type females fed 8×S diet (p < 0.05; Figure 3C). Importantly, 8×S did not repress these transcripts in *dfoxoΔ* flies (p = 6 × 10^−4^ for genotype by diet interaction; Figure 3C), confirming diet-induced changes as mediated by dFOXO inhibition. Hence, sugar-rich diet induces *dilp6* and inhibits dFOXO to repress dFOXO target genes, including epigenetic modifiers.

The induction of *dilp6*, phosphorylation of dFOXO, and repression of its targets did not persist after recovery on 1×S diet (Figures 3A, 3B, and S2A). However, the transient repression of dFOXO may have long-term consequences through the observed, *dfoxo*-dependent regulation of epigenetic modifiers. To find evidence of long-term changes to chromatin, we examined nuclear DNA distribution in the fat body, an adipose-like organ in which dFOXO activity extends lifespan (Giannakou et al., 2004, Hwangbo et al., 2004). Nuclear DNA distribution is indicative of global chromatin arrangements (Fedorova and Zink, 2009, Tian et al., 2016). The frequency of DNA foci staining brightly with DAPI was increased after recovery from 8×S in the wild-type, but not *dfoxoΔ*, flies, which already displayed high levels on 1×S (Figure 3D), consistent with dfoxo-dependent resetting of chromatin states by 8×S. Such changes in chromatin states would be expected to result in persistent transcriptional changes. To examine whether any such changes exist, we performed RNA-seq on flies that consumed 8×S or 1×S food for 1 week and then recovered on 1×S for 1 week. This experiment was performed at the same time as analysis of transcriptional changes during 8×S feeding described above, allowing us to compare expression both before and after recovery. For an unbiased assessment of global transcriptional changes, we performed principal-component analysis (PCA) on all samples. PCA suggested an overall difference in the transcriptomes of flies that had recovered from the 8×S diet and their sisters, which were continuously kept on 1×S, indicating there is a long-term transcriptional program set up by 8×S feeding (Figure 3E). This program was distinct from the response observed before recovery both globally (Figure 3E) and at the level of differential expression of individual genes (Figure S2B; Data S1), indicating it is not simply a carryover of the changes occurring before recovery. Hence, feeding with 8×S appears to place a novel, historic signature upon the fly’s transcriptome.

Genes differentially expressed after recovery were not enriched for forkhead-like motifs in their promoters or for genes differentially expressed in *dfoxo* nulls, consistent with cessation of dFOXO repression after recovery. To address whether *dfoxo* is required for the transcriptional legacy of the 8×S diet, we profiled the mRNA levels of the gene encoding glycine N-methyltransferase (*Gnmt*), chosen because it was the most highly induced gene in response to 8×S after recovery (Figure S2B). We confirmed that *Gnmt* mRNA levels were significantly induced by prior exposure to 8×S diet after 1 week of recovery in wild-type flies (p < 0.05; Figure 3F). *Gnmt* could simply be responding to the long-term changes in the levels of its substrate, S-adenosyl-methionine (SAM). However, we found that, even though SAM levels increased on 8×S diet, the increase did not persist and could not account for increased *Gnmt* levels after recovery (Figure S2C). Hence, *Gnmt* induction is a transcriptional marker of the history of high-sugar feeding independent of current SAM levels. Importantly, *Gnmt* induction was not observed in *dfoxoΔ* females (p = 0.03 for genotype by sugar interaction; Figure 3F), revealing that *dfoxo* is required for this transcriptional memory of past sugar consumption. Overall, our data imply that a high-sugar diet reversibly inhibits dFOXO to cause short- and long-term transcriptional changes, perhaps accounting for the programming of lifespan observed.

### *dfoxo*/*daf-16* Are Required for Programming of Lifespan by Early-Life Sugar-Rich Diet

**Figure 4:**
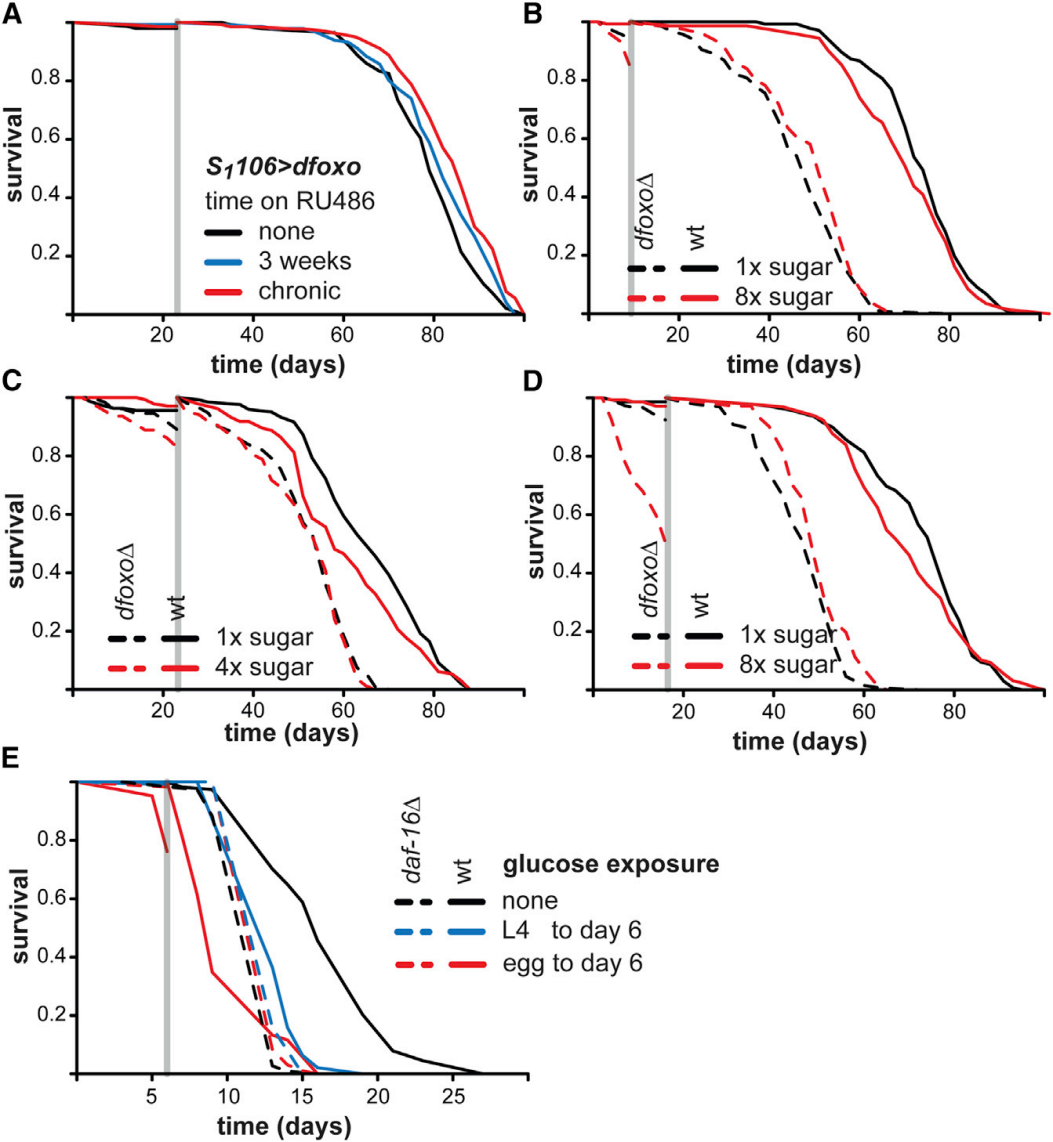
*dfoxo* and *daf-16* Are Required for Late-Life Detrimental Effects of Early-Life Diet High in Sugar (A) *dfoxo* was induced in the gut/fat body of adult females with the *S*_*1*_*106* driver from day 2 of adulthood, either chronically or for 3 weeks. Both acute and chronic *dfoxo* overexpression extended lifespan after day 23 (p < 0.05; log rank test). (B–D) Survival of wild-type or *dfoxoΔ* females after feeding on 8×S for 1 week (B), 4×S for 3 weeks (C), or 8×S for 2 weeks (D) compared to those continuously kept on 1×S. (E) Survival of wild-type or *daf-16* worms after treatment with glucose starting from either embryo (egg) or L4 and lasting up to day 6 of adulthood. In all panels, the gray vertical bar indicates the time of switch, when survival was reset to 1. See also Tables S2–S5.

Our data suggested that altered dFOXO activity in early life can influence subsequent survival. Indeed, 3-week induction of *dfoxo* in the gut and fat body of adult females using the tissue-specific, inducible, *S*_*1*_*106* driver was sufficient to extend their subsequent lifespan (p < 0.05; Figure 4A; Giannakou et al., 2007). Importantly, dFOXO levels have been shown to revert back to normal after induction ceases (Giannakou et al., 2007). Whereas the lifespan effect of transient induction was less than achieved by chronic induction (Figure 4A), it confirmed that dFOXO activity in early life can have long-term consequences.

Is *dfoxo* required for the long-term, detrimental effect of a sugar-rich diet? We tested this in three independent experiments. *dfoxoΔ* females are short lived, both on 1×S and 8×S (Al Saud et al., 2015). To avoid bias that could result from a strong selection on the *dfoxoΔ* population, we limited the exposure to 8×S in early adulthood to only 1 week in the first experiment (Figure 4B). In the second, we limited the dose of sugar to 4×S for 3 weeks (Figure 4C). Finally, we tested whether 2 weeks on 8×S, which resulted in nearly 50% of the *dfoxoΔ* population dying during treatment, could elicit a response in the surviving *dfoxoΔ* flies (Figure 4D). We assessed survival after treatment to isolate the effects of dietary history. Transient feeding with sugar-rich diets and the deletion of *dfoxo* both had a significant, detrimental effect on subsequent survival (p < 2 × 10^−16^ in all experiments; Tables S2–S4). Importantly, historical exposure to sugar-rich diets did not further shorten the lifespan of *dfoxoΔ* females in any experiment (p < 0.05 for interaction between diet and genotype; Figures 4B–4D; Tables S2–S4), revealing that *dfoxo* is required for the effects of dietary history on survival. This implies that a high-sugar diet in early adulthood acts through *dfoxo* to program lifespan.

We sought to establish whether the role of dFOXO in nutritional programming of lifespan was conserved in its worm ortholog, DAF-16. Chronic exposure to glucose reduces worm lifespan by inhibiting DAF-16 (Lee et al., 2009, Schulz et al., 2007), but the effects of transient exposure have not been examined. Presence of additional glucose in the media throughout development or during the first 6 days of adulthood (a third of median life expectancy) reduced the subsequent survival in wild-type worms (p < 2 × 10^−16^; Figure 4E; Table S5), revealing a lasting detrimental effect equivalent to that in the fly. Importantly, the survival of the *daf-16* worms was not sensitive to glucose in early life (Figure 4E; p < 2 × 10^−16^ for interaction between diet and genotype; Table S5). Hence, the role of FOXO factors in mediating nutritional programming of lifespan is evolutionarily conserved between flies and worms, making it likely that they play an equivalent role in nutritional programming in mammals.

## Discussion

Epidemiological and other data have provided extensive evidence that nutrition in early life can have lasting consequences for aging and age-related disease in mammals, including humans (Alfaradhi and Ozanne, 2011, Barnes and Ozanne, 2011). However, to date, it has been unclear whether any of the several evolutionarily conserved, longevity-assurance mechanisms that have been discovered in simpler animal models (Gems and Partridge, 2013) connect early nutrition to health and survival in later life. Our study strongly implicates FOXO factors as this missing mechanistic link between early-life nutrition and longevity. The strong evolutionary conservation of FOXO function makes it highly likely that FOXO factors play a role in some aspect of nutritional programming in mammals.

The role of dFOXO in mediating the long-term effects of a sugar-rich diet in *Drosophila* is surprisingly specific. dFOXO is not required for the lifespan benefits of a chronic reduction in protein intake, even though its activity can modulate the response (Giannakou et al., 2008). Similarly, the survival of *dfoxo*-null flies is reduced by chronic feeding with a sugar-rich diet to the same extent as the wild-type’s (Al Saud et al., 2015). These differences in the role of dFOXO in response to chronic or acute dietary regimes, or different dietary components, may arise from the complex interactions between nutrition and insulin/IGF-like signaling: each DILP is expressed in a unique tissue pattern, acts in an endocrine and/or paracrine manner, and responds distinctly to the relative amounts of protein and carbohydrate present in the diet (Post and Tatar, 2016). This, in turn, may specify the tissues in which dFOXO is inhibited by specific diets and the nature of dFOXO targets affected.

In aging studies, the principal focus is on discovering and understanding mechanisms whereby lifespan can be extended and health maintained in later life. Several instances already exist where a transient intervention, be it during development or in adulthood, can have prolonged beneficial consequences (Bitto et al., 2016, Dillin et al., 2002, Schulz et al., 2007). For example, mild impairment of mitochondrial function during worm development can extend adult lifespan (Dillin et al., 2002), and these long-term effects are mediated by epigenetic changes (Merkwirth et al., 2016, Tian et al., 2016). We have investigated how lifespan is curtailed, rather than extended, by unhealthy nutrition in early adulthood. Similar to transient, lifespan-extending interventions, it is likely that the long-term, detrimental effects of diet-induced FOXO inhibition are also due to persistent epigenetic modifications. Indeed, DAF-16 engages the SWI/SNF chromatin-remodeling complex to increase worm lifespan (Riedel et al., 2013). Our gene expression data show that, in *Drosophila*, a sugar-rich diet represses dFOXO to drive changes in expression of a number of epigenetic modifiers, which is likely to have substantial consequences for the epigenome. Elucidating these *dfoxo*-dependent epigenetic changes and how they could be reversed may form the basis of future treatments to remedy the cost of past diets.

## Experimental Procedures

### Fly Husbandry, Food, Feeding, and Lifespan Assays

Outbred, Dahomey fly population carrying the *w*^*1118*^ mutation was used in all experiments. *D*. *melanogaster* diet contained 10% yeast, 1.5% agar with 40% (8×S) or 5% (1×S) sucrose (all w/v). When required, RU486 (200 μM) was included in the food. *C*. *elegans* were reared as per Brenner (1974) and worms exposed to 2% glucose in NGM plates from embryo or from L4 stage. RNA was isolated with Trizol for qPCR and RNA sequencing. Protein samples were extracted in TCA and dFOXO phosphorylation assessed as described (Alic et al., 2011, Giannakou et al., 2007). Transcriptomes were analyzed by aligning reads to *Drosophila* genome dm6 in Tophat2, enumerating reads with HTSeq, and model fitting using DESeq2. TF-binding motif enrichment was analyzed using iRegulon. Survival was analyzed in R and JMP. See Supplemental Experimental Procedures for full details of animal husbandry, molecular, and data analysis.

## Author Contributions

N.A. devised the study; A.J.D., M.E., C.E.F., and A.C.S. performed experiments; A.J.D., M.E., C.E.F., A.C.S., and N.A. analyzed the data; M.D.W.P., D.G., and N.A. supervised the study; and A.J.D., M.E., M.D.W.P., D.G., and N.A. wrote the manuscript.

## Accession Numbers

The accession number for the RNA-seq data reported in this paper is ArrayExpress: E-MTAB-4766.

## Supplemental Information

Document S1. Supplemental Experimental Procedures, Figures S1 and S2, and Tables S1–S5

Data S1. Supplementary Data Files, Related to Figures 2 and 3

Document S2. Article plus Supplemental Information
