# Supplementary material for: Nutritional Programming of Lifespan by FOXO Inhibition on Sugar-Rich Diets
Source: Cell Rep. 2017 Jan 10;18(2):299–306. doi: 10.1016/j.celrep.2016.12.029 (PMC5263231; doi:10.1016/j.celrep.2016.12.029)
Supplement: Document S1. Supplemental Experimental Procedures, Figures S1 and S2, and Tables S1–S5 [file mmc1.pdf]

Cell Reports, Volume *18*

Supplemental Information

Nutritional Programming of Lifespan by FOXO Inhibition on Sugar-Rich Diets

Adam J. Dobson, Marina Ezcurra, Charlotte E. Flanagan, Adam C. Summerfield, Matthew D.W. Piper, David Gems, and Nazif Alic

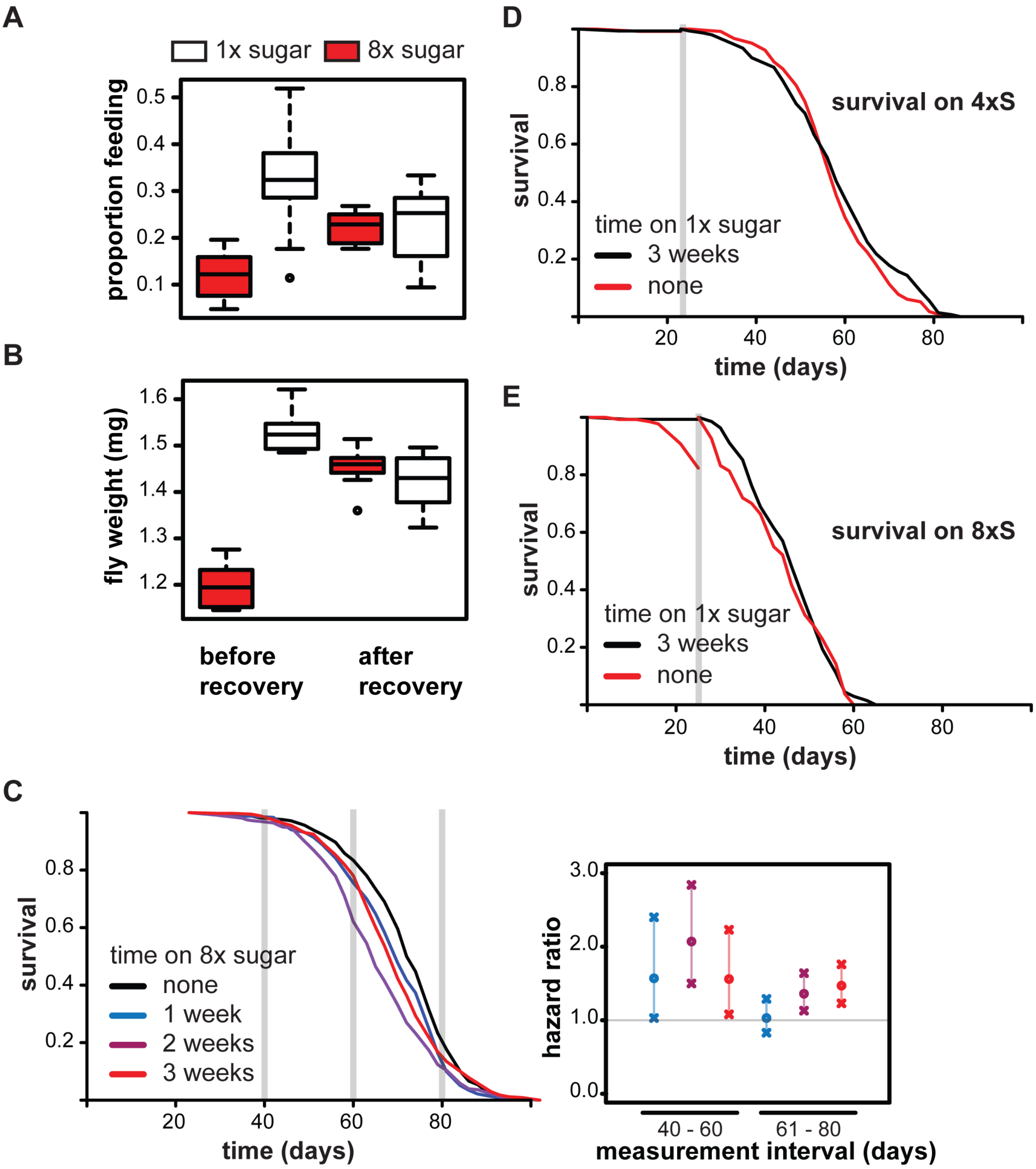

Figure S1. Related to Figure 1. Wild-type flies were fed 1xS or 8xS diet for a week and then allowed to recover on 1xS diet for a week. Proportion of flies feeding (**A**) and fly weight (**B**) were determined at the end of the first week (before recovery) and at the end of the second week (after recovery). Both feeding rate and body weight were significantly reduced by 8xS before but not after recovery (Body weight ANOVA diet * time interaction, $p = 6x10^{-9}$; Proportion feeding GLM diet * time interaction, $p=8x10^{-4}$). **C** Survival curves pooled from multiple experiments (including those in the main figures) and the hazard ratios and 95% confidence intervals calculated from events in the indicated time periods. Total number of dead/censored flies: control: 651/22; 1 week treatment: 233/9 ; 2 weeks: 359/3; 3 weeks: 357/4. To calculate the hazard ratios a mixed-effect CPH model was fitted with experimental trial as a random effect. **D** and **E** – Survival on diets rich in sugar after 3 weeks (23 days for G) on 1xS compered to no exposure to 1xS. Time on 1xS did not significantly increase survival.

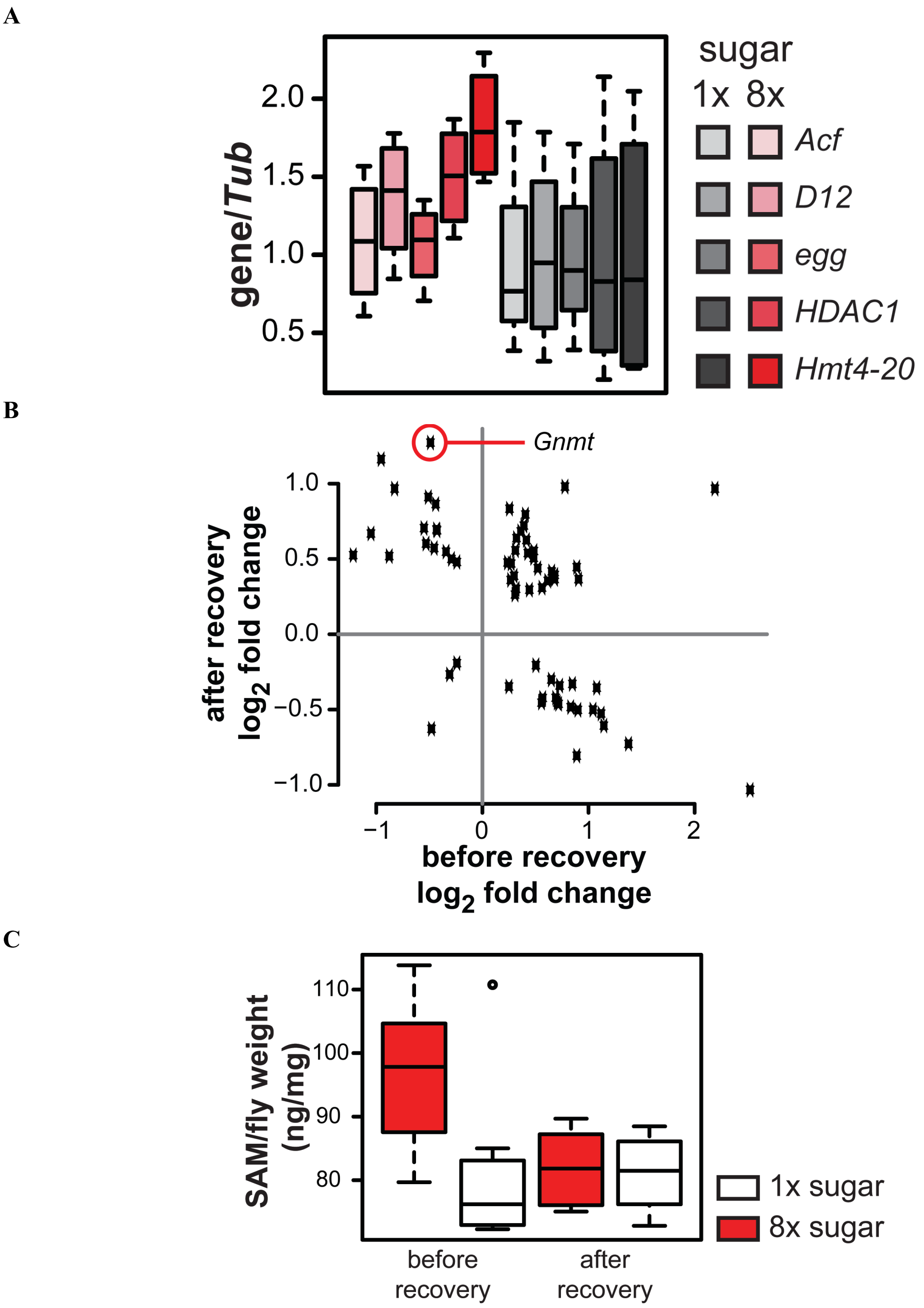

Figure S2. Related to Figure 3. A Expression of *Acf*, *D12*, *egg*, *HDAC1* and *Hmt4-20* after recovery in the wild-type. Data were scaled to 1xS and analysis with a liner model indicated a near-significant induction of all transcripts by 8xS after recovery ($p=0.06$). **B** Log_2 fold expression ~~change~~ induced by 8xS before and after recovery for the set of genes differentially expressed after recovery (10% FDR), with *Gnmt* indicated. **C** Levels of SAM before and after a week of recovery following from a week of treatment with indicated food. Data were analysed with a linear model, revealing a significant effect of sugar and time ($p<0.05$) and a significant interaction of the two ($p=0.01$) where SAM levels were elevated in response to 8xS before ($p<0.05$, t-test) but not after recovery.

Table S1. Related to Figure 1B. CPH analysis of survival data.

**coefficient***	**estimate****	**s.e.**	z	***p*-value**
time on 8x sugar	4.2	0.22	18.82	$<2x10^{-16}$
decay of sugar effect	-0.084	0.0046	-18.23	$<2x10^{-16}$

*Exposure to sugar was modeled as duration of exposure in weeks, the decay of the sugar effect was modeled as the interaction between the effect of sugar and time since exposure in days. **The estimate is the logarithm of the relative risk, where a positive value indicates an increase in relative risk of death. Total flies dead = 389, censored = 11.

Table S2. Related to Figure 4B. CPH analysis of survival data.

**coefficient***	**estimate****	**s.e.**	z	***p*-value**
time on 8x sugar	9.1	0.55	17	$<2x10^{-16}$
decay of sugar effect	-0.13	$8.1x10^{-3}$	-16	$<2x10^{-16}$
foxoΔ	3.3	0.16	20	$<2x10^{-16}$
foxoΔ : time on 8x sugar	-2.1	0.66	-3.2	$1.4x10^{-3}$
foxoΔ : decay of sugar effect	-0.023	0.98	-1.8	0.077

*Time of exposure to 8x sugar was expressed in weeks, the decay of the sugar effect was modeled as the interaction between the effect of sugar and time since exposure in days, ”:” indicates an interaction term.

**The estimate is the logarithm of the relative risk, where a positive value indicates an increase in relative risk of death. Total flies dead = 533, censored = 19.

Table S3. Related to Figure 4C. CPH analysis of survival data.

**coefficient***	**estimate****	**s.e.**	***z***	***p*-value**
time on 4x sugar	2.6	0.15	17	$<2x10^{-16}$
decay of sugar effect	-0.053	$3.2x10^{-3}$	-16	$<2x10^{-16}$
foxoΔ	2.2	0.18	12	$<2x10^{-16}$
foxoΔ : time on 4x sugar	-0.4	0.17	-2.4	0.018
foxoΔ : decay of sugar effect	-0.011	$4.7x10^{-3}$	-2.4	0.019

*Time of exposure to 4x sugar was expressed in weeks, the decay of the sugar effect was modelled as the interaction between the effect of sugar and time since exposure in days, ":" indicates an interaction term.
**The estimate is the logarithm of the relative risk, where a positive value indicates an increase in relative risk of death.
Total flies dead = 434, censored = 26.

Table S4. Related to Figure 4D. CPH analysis of survival data.

**coefficient***	**estimate****	**s.e.**	*z*	***p*-value**
time on 8x sugar	4.4	0.28	15	$<2x10^{-16}$
decay of sugar effect	-0.07	$4.5x10^{-3}$	-15	$<2x10^{-16}$
foxoΔ	4	0.21	19	$<2x10^{-16}$
foxoΔ : time on 8x sugar	-1.4	0.41	-3.6	$3.5x10^{-4}$
foxoΔ : decay of sugar effect	-0.014	$9.7x10^{-3}$	-1.4	0.16

*Time of exposure to 8x sugar was expressed in weeks, the decay of the sugar effect was modelled as the interaction between the effect of sugar and time since exposure in days, ":" indicates an interaction term.

**The estimate is the logarithm of the relative risk, where a positive value indicates an increase in relative risk of death. Total flies dead = 469, censored = 7.

Table S5. Related to Figure 4E. CPH analysis of survival data.

**Start of treament***	**coefficient****	**estimate*****	**s.e.**	***z***	***p*-value**
embryo (egg)	glucose	2.2	0.2	11	$<2x10^{-16}$
	daf-16	1.9	0.18	11	$<2x10^{-16}$
	glucose : *daf-16*	-2.4	0.24	-10	$<2x10^{-16}$
L4	glucose	1.4	0.17	8.4	$<2x10^{-16}$
	daf-16	2.2	0.18	13	$<2x10^{-16}$
	glucose : *daf-16*	-1.8	0.16	-8.3	$<2x10^{-16}$

*Separate CPH models were fitted using the same control (no glucose) data.
**Glucose was fitted as categorical covariate, ":" indicates an interaction term.
***The estimate is the logarithm of the relative risk, where a positive value indicates an increase in relative risk of death.
Total worms dead/censored: from embryo (egg) = 379/23, from L4 = 417/20.

Extended experimental procedures

Fly husbandry, food, feeding and lifespan assays

dfoxoΔ^{94} mutant (Slack et al., 2011), S_1106 and *UAS-dfoxo* (Giannakou et al., 2004) were backcrossed at least 6 times into the wild-type, outbred, Dahomey population carrying the w^{1118} mutation that was used in all experiments. The Dahomey stock was collected in 1970 in Dahomey (now Benin) and kept in population cages to maintain its lifespan and fecundity at levels similar to freshly caught stocks. The lines were maintained, and all experiments performed, at 25ºC with 60% humidity and 12h:12h light:dark cycle. Flies were maintained on food containing 10% yeast, 5% sucrose, 1.5% agar (all w/v; 1xS food) (Bass et al., 2007). Experimental flies were reared from embryo at standardised densities on 1xS food and females were sorted at random onto the appropriate food 48h after emergence and *ad libitum* mating. 8xS food contained 10% yeast, 40% sucrose, 1.5% agar. Lifespan experiments were performed as described previously (Al Saud et al., 2015) on cohorts of flies housed 10 females per vial. Feeding rate was measured by a proboscis extension assay, counting the number of flies feeding per instantaneous observation per vial, every 5 minutes for 2 hours between 1h and 3h into the light cycle, after overnight acclimatisation to the observation arena.

Worm husbandry, food and lifespans assays

Standard *C. elegans* culture was as previously described (Brenner, 1974). Strains were grown at 20°C on NGM plates seeded with *E. coli* OP50. N2 (wild type) and GR1307 *daf-16(mgDf50)* were used. For glucose experiments, 2% glucose was added to molten NGM before plate pouring. Survival assays were performed at 20°C on plates supplemented with 5-Fluoro-2'-deoxyuridine to 15µM to inhibit progeny growth.

RNA, qPCR, protein extractions, western blots, weight and SAM measurements, DAPI staining

10 females per sample were snap-frozen in liquid nitrogen. RNA was extracted with Trizol, converted to cDNA and qPCR performed using *dilp6* primers (Gronke et al., 2010) or the following primers:

Primers used for qPCR

Primer ID (gene, Fwd/Rev)	*Sequence*
Acf F	CGCGACTATGAACACTAC
Acf R	TCCTCGTAGGTGAGGTTC
D12 F	CTAATGCACACGGTGGTG
D12 R	ATCGCCTGTTTTGCTCTCAG
egg F	GCTGCGTGTCCCAAGACG

egg R	GGGCAAAGGCACGCATCTG
Gnmt F	GGAGGCGTCCTGCTTATC
Gnmt R	CCGTGTGACTCGTATTATAG
HDAC1 F	GTTTGTTACTACTACGACAGC
HDAC1 R	CGATAGAGCCCATAGTTG
Hmt4-20 F	CGGCTCCACGATCATATC
Hmt4-20 R	CCCGTTGCTCTTCCAGTG
Tubulin F	TGGGCCCGTCTGGACCACAA
Tubulin R	TCGCCGTCACCGGAGTCCAT

Proteins were extracted with TCA, separated by SDS-PAGE and transferred to nitrocellulose membranes and visualised using the antibody previously described (Alic et al., 2011; Giannakou et al., 2007). The slower migrating form has been shown to correspond to phosphorylated dFOXO (Alic et al., 2011).

For body weight and SAM assays, flies were CO_2 anaesthetised and weighed, then flash-frozen in liquid N_2. They were thawed on ice, homogenized with a micropestle in 75 µl ice-cold PBS, and SAM was assayed using the SAM Elisa Kit (Cell Biolabs STA-672) as per the manufacturer's instructions.

For DAPI staining, abdominal fat bodies, as associated with the cuticle, were dissected and fixed with 4% formaldehyde, washed and stained with DAPI. Confocal stacks were obtained on Zeiss LSM700. The number of bright spots per nucleus was determined in Volocity (PerkinElmer).

RNA sequencing and analysis

RNA sequencing (paired-end, 75bp per read) of poly-A RNA was performed by Glasgow Polyomics. The raw data have been submitted to Array Express (E-MTAB-4766). The reads were assessed for quality using FastQC, and aligned to Berkeley Drosophila Genome Project assembly release 6, where genes with exon overlap of >50% were combined into a pseudo-feature, and non-chromosomal regions excluded, using TopHat2. After converting BAM files to SAM files using SAMTOOLS, reads were counted using HTSeq (Anders et al., 2013). Four biological repeats were sequenced per treatment (one sample from the 8xS diet and its correspondent from the 1xS diet, both before recovery, were removed from subsequent analysis following preliminary assessment with PCA). Genes were filtered from differential expression analysis if ≤1 read was detected across all samples. Differential expression was analysed in R using a generalized linear model (GLM) in DESeq2 (Anders and Huber, 2010) using a beta prior. The GLM included each dietary condition (1 week of

exposure to 8xS/1xS, with/without 1 week of subsequent recovery on 1xS) as an unordered factor, and biological replicate as a cofactor. P values were adjusted using independent hypothesis weighting (Ignatiadis et al., 2016) with a significance threshold (alpha) of 0.1. Fold-changes in expression were calculated by the internal DESeq functions. *dfoxo* differential expression was as determined by (Alic et al., 2011) but at an FDR threshold of 0.1 to match the present study. Overlap between the sugar-responsive and *dfoxo*-responsive gene sets was determined by a hypergeometric test using the phyper() function in R. Principal components analysis was performed using the prcomp() function in R, on variance-stabilised reads (whole transcriptome) produced by DESeq2. GO enrichment was assessed with topGO (Alexa A and Rahnenfuhrer J (2016). topGO: Enrichment Analysis for Gene Ontology. R package version 2.24.0.) using a custom GO annotation accounting for pseudofeatures in the gtf file. Enrichment of GO terms was performed using the "weight01" algorithm to account for GO term structure, and applying Fisher's test for enrichment. Enrichment of transcription factor-binding motifs was analysed with iRegulon (Janky et al., 2014), using default parameters, and excluding features which are not annotated to bind known TFs. ~~Raw data have been deposited in ArrayExpress under the accession number: E-MTAB-4766.~~ The counts table, gene lists, outputs of GO and TF-binding site analysis are given in the **Supplemental Data**.

Statistical analysis

Survival data was analysed with Cox Proportional Hazards (CPH) in R using the "survival" package (Terry Therneau, http://CRAN.R-project.org/package=survival). Time-dependent covariates were dealt with as recommended by (Fox, 2008): the decay of the sugar effect was modelled as an interaction between the effect of sugar and time since end of treatment (time on 8x (weeks) : time since end of exposure (days)) so that the models described in supplemental tables took the form: Survival ~ time on 8x (weeks) + genotype + decay of sugar effect + genotype : time on 8x (weeks) + genotype : decay of sugar effect, where ":" specifies an interaction. For assessment of survival in specified intervals, all events occurring after the interval were included as censors and effects estimated using CHP: Survival ~ time on 8x (categorical) + experimental trial (random effect). qPCR data were scaled and analysed with a linear model in JMP (version 11) software (SAS Institute), followed by planned, pair-wise t-tests. Similarly, analysis of western blot quantification was performed with a mixed-effect linear model. Number of DAPI foci per nucleus was analysed with a generalised linear model with Poisson distribution in JMP. Log-rank test was performed in JMP. Details of statistical tests are given in figure and table captions.

Supplemental References

Al Saud, S., Summerfield, A., and Alic, N. (2015). Ablation of insulin-producing cells prevents obesity but not premature mortality caused by a high-sugar diet in *Drosophila*. Proc R Soc B *282*, 20141720.

Alic, N., Andrews, T.D., Giannakou, M.E., Papatheodorou, I., Slack, C., Hoddinott, M.P., Cocheme, H.M., Schuster, E.F., Thornton, J.M., and Partridge, L. (2011). Genome-wide dFOXO targets and topology of the transcriptomic response to stress and insulin signalling. Molecular Systems Biology *7*.

Anders, S., and Huber, W. (2010). Differential expression analysis for sequence count data. Genome Biol *11*, R106.

Anders, S., McCarthy, D.J., Chen, Y., Okoniewski, M., Smyth, G.K., Huber, W., and Robinson, M.D. (2013). Count-based differential expression analysis of RNA sequencing data using R and Bioconductor. Nat Protoc *8*, 1765-1786.

Bass, T.M., Grandison, R.C., Wong, R., Martinez, P., Partridge, L., and Piper, M.D. (2007). Optimization of dietary restriction protocols in Drosophila. J Gerontol A Biol Sci Med Sci *62*, 1071-1081.

Brenner, S. (1974). The genetics of Caenorhabditis elegans. Genetics *77*, 71-94.

Fox, J. (2008). Cox proportional-hazards regression for survival data. Appendix to An R and S-Plus Companion to Applied Regression.

Giannakou, M.E., Goss, M., Jacobson, J., Vinti, G., Leevers, S.J., and Partridge, L. (2007). Dynamics of the action of dFOXO on adult mortality in Drosophila. Aging Cell *6*, 429-438.

Giannakou, M.E., Goss, M., Junger, M.A., Hafen, E., Leevers, S.J., and Partridge, L. (2004). Long-lived Drosophila with overexpressed dFOXO in adult fat body. Science *305*, 361.

Gronke, S., Clarke, D.-F., Broughton, S., Andrews, T.D., and Partridge, L. (2010). Molecular evolution and functional characterisation of Drosophila insulin-like peptides. PLoS Genet *6*, e1000857.

Ignatiadis, N., Klaus, B., Zaugg, J.B., and Huber, W. (2016). Data-driven hypothesis weighting increases detection power in genome-scale multiple testing. Nature methods *13*, 577-580.

Janky, R., Verfaillie, A., Imrichova, H., Van de Sande, B., Standaert, L., Christiaens, V., Hulselmans, G., Herten, K., Naval Sanchez, M., Potier, D., et al. (2014). iRegulon: from a gene list to a gene regulatory network using large motif and track collections. PLoS computational biology *10*, e1003731.

Slack, C., Giannakou, M.E., Foley, A., Goss, M., and Partridge, L. (2011). dFOXO-independent effects of reduced insulin-like signaling in Drosophila. Aging Cell *10*, 735-748.
