# Supplementary material for: Nutritional Programming of Lifespan by FOXO Inhibition on Sugar-Rich Diets
Source: Cell Rep. 2017 Jan 10;18(2):299–306. doi: 10.1016/j.celrep.2016.12.029 (PMC5263231; doi:10.1016/j.celrep.2016.12.029)
Supplement: Document S2. Article plus Supplemental Information [file mmc3.pdf]

Cell Reports

Report

Nutritional Programming of Lifespan by FOXO Inhibition on Sugar-Rich Diets

Graphical Abstract

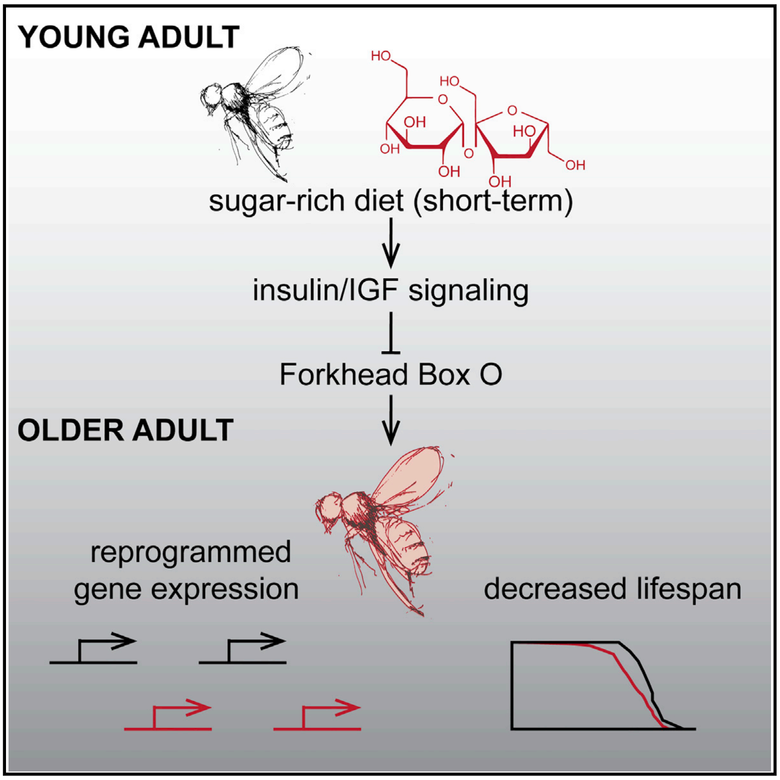

Highlights

- A high-sugar diet in early life programs *Drosophila* lifespan
- High sugar inactivates dFOXO, altering mRNA levels of chromatin modifiers
- *dfoxo* is required for long-term transcriptional changes in response to high sugar
- *dfoxo* and *daf-16* are required for programming of lifespan by high-sugar diets

Authors

Adam J. Dobson, Marina Ezcurra, Charlotte E. Flanagan, Adam C. Summerfield, Matthew D.W. Piper, David Gems, Nazif Alic

Correspondence

n.alic@ucl.ac.uk

In Brief

Modern diets can have negative consequences for long-term health. Dobson et al. show that high-sugar diets program fly and worm lifespan through the regulation of forkhead box O transcription factors.

Accession Numbers

E-MTAB-4766

Dobson et al., 2017, Cell Reports *18*, 299–306
January 10, 2017 © 2017 The Author(s).
http://dx.doi.org/10.1016/j.celrep.2016.12.029

Cell Reports
Report

OPEN ACCESS
CellPress

Nutritional Programming of Lifespan by FOXO Inhibition on Sugar-Rich Diets

Adam J. Dobson,[1] Marina Ezcurra,[1] Charlotte E. Flanagan,[1,3] Adam C. Summerfield,[1,4] Matthew D.W. Piper,[2] David Gems,[1] and Nazif Alic[1,5,*]
[1]Institute of Healthy Ageing and Department of Genetics, Evolution and Environment, University College London, Gower Street, WC1E 6BT London, UK
[2]School of Biological Sciences, Monash University, Melbourne, VIC 3800, Australia
[3]Present address: Faculty of Life Sciences and Medicine, King's College London, 2nd Floor Borough Wing, Guy's Hospital, Great Maze Pond, SE1 9RT London, UK
[4]Present address: Center for Molecular Biomedicine, Institute for Biochemistry and Biophysics, Department of Biochemistry, Friedrich Schiller University of Jena, Hans-Knöll-Straße 2, 07745 Jena, Germany
[5]Lead Contact
*Correspondence: n.alic@ucl.ac.uk
http://dx.doi.org/10.1016/j.celrep.2016.12.029

SUMMARY

Consumption of unhealthy diets is exacerbating the burden of age-related ill health in aging populations. Such diets can program mammalian physiology to cause long-term, detrimental effects. Here, we show that, in *Drosophila melanogaster*, an unhealthy, high-sugar diet in early adulthood programs lifespan to curtail later-life survival despite subsequent dietary improvement. Excess dietary sugar promotes insulin-like signaling, inhibits dFOXO—the *Drosophila* homolog of forkhead box O (FOXO) transcription factors—and represses expression of dFOXO target genes encoding epigenetic regulators. Crucially, *dfoxo* is required both for transcriptional changes that mark the fly's dietary history and for nutritional programming of lifespan by excess dietary sugar, and this mechanism is conserved in *Caenorhabditis elegans*. Our study implicates FOXO factors, the evolutionarily conserved determinants of animal longevity, in the mechanisms of nutritional programming of animal lifespan.

INTRODUCTION

Age is the main risk factor for a plethora of chronic human illnesses (Niccoli and Partridge, 2012). Aging is influenced by many parameters throughout the life of an individual, with most variation in human lifespan attributable to environmental differences (Slagboom et al., 2011). Currently, one of the main environmental insults on human health is the food we eat (Lustig et al., 2012). Indeed, chronic diseases are on the rise globally, due in part to aging populations (Christensen et al., 2009) but also due to the increasing consumption of unhealthy diets dominated by highly processed, low-cost foods (Dearden and Ozanne, 2015; Lustig et al., 2012). For example, sugar consumption has tripled over the last 50 years and is linked to a range of detrimental health outcomes (Lustig et al., 2012). These conditions underlie a pandemic of metabolic disorders, such as obesity and diabetes, which amplify the disease burden of an increasingly aged population.

An individual's long-term adult health is influenced not only by their current diet but also by dietary history. Persistent effects of nutrition are termed nutritional programming, where a nutritional stimulus triggers a structural change or a persistent physiological state with long-term functional consequences (Lucas, 1998). In humans and other mammals, there is a wealth of epidemiological and experimental evidence that both under- and over-nutrition in early life can profoundly influence later-life health and survival. Long-term effects arise during development, potentially in early adulthood, and can be transmitted from parent to offspring (Dearden and Ozanne, 2015; Fernandez-Twinn et al., 2014; Gillman, 2005; Hardikar et al., 2015; Hirko et al., 2015; Langley-Evans, 2006). Importantly, even small, persistent effects can have important societal consequences due to the sheer number of people consuming unhealthy diets (Lustig et al., 2012). The molecular mechanisms that link past nutritional experience to curtailed survival and detrimental health outcomes remain unclear.

Drosophila melanogaster is an important model in understanding the interaction between nutrition and aging (Piper et al., 2011; Simpson et al., 2015; Tatar et al., 2014). To be consistent with their ecology, laboratory fruit flies are often fed a diet composed of yeast (a protein source) and sugar (Bass et al., 2007). High-protein diets shorten *Drosophila* lifespan (Lee et al., 2008; Partridge et al., 1987; Skorupa et al., 2008), but the effects of protein excess on mortality appear completely and rapidly reversible in adult *Drosophila* (Mair et al., 2003), indicating that protein-skewed diets do not program subsequent mortality (Mair et al., 2003). However, some diet-induced physiological changes are irreversible in the adult fly (van den Heuvel et al., 2014). In fruit flies, increased mortality due to excessive dietary sugar continues after dietary change (Mair et al., 2005), whereas parental sugar consumption enhances obese-like phenotypes in the offspring (Buescher et al., 2013; Öst et al., 2014). These studies hint that sugar-rich diets can program *Drosophila* physiology and

This is an open access article under the CC BY license (http://creativecommons.org/licenses/by/4.0/).

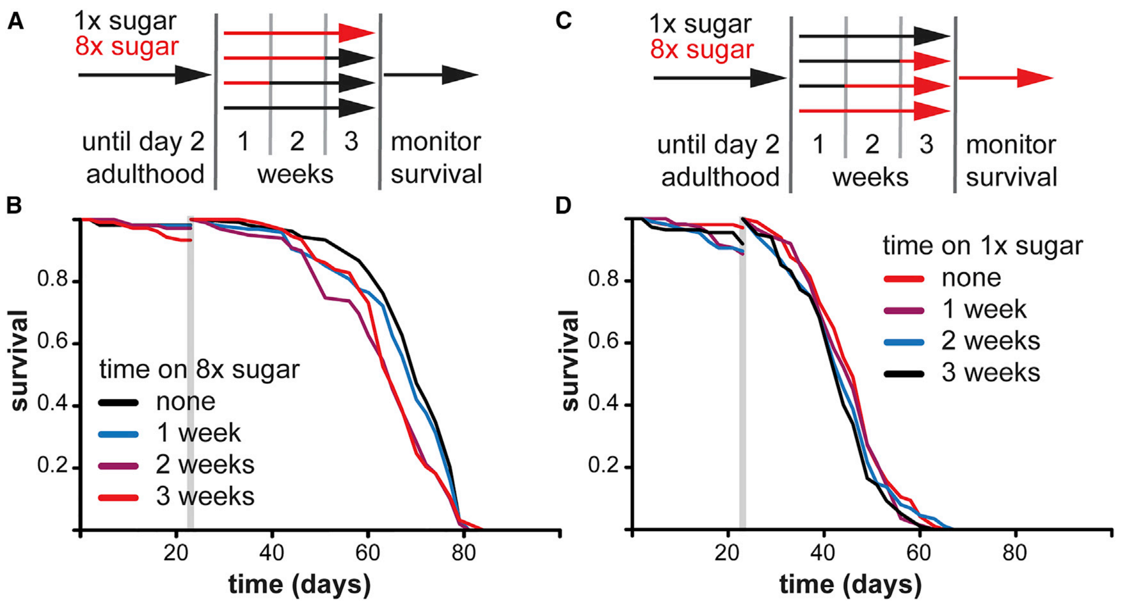

Figure 1. Excess Sugar in Early Adulthood Curtails Later-Life Survival

(A) Experimental design.

(B) Survival of females after feeding on 8×S for 1–3 weeks, compared to those continuously kept on 1×S.

(C) Experimental design of the reverse switch.

(D) Survival of females on 8×S after feeding on 1×S for 1–3 weeks compared to those continuously kept on 8×S. Total dead = 403; censored = 46. Only 3 weeks on 1×S showed a significant difference to control (reduced survival; p = 0.02; log rank test).

In both (B) and (D), the gray vertical bar indicates the time of the last switch (23 days), when survival was reset to 1. See also Figure S1 and Table S1.

should be explored as a model of the mechanisms connecting dietary history to aging.

Here, we show that consuming a diet high in sugar (sucrose) in early adulthood curtails later-life survival in *Drosophila* through nutritional programming. We show that sugar regulates the activity of the *Drosophila* forkhead box O (FOXO) transcription factor (TF) to set up gene expression changes that mark the fly's nutritional history. We find that *dfoxo* is required to establish long-term, detrimental effects of past excessive sugar consumption. Importantly, this role of *dfoxo* is conserved in its *Caenorhabditis elegans* ortholog, *daf-16*. Our findings reveal FOXO factors as a mechanistic link between dietary history and later-life survival.

RESULTS

Excessive Sugar in Early Adult Fly Diet Curtails Survival in Middle and Old Age

Can excess sugar in the adult diet program *Drosophila* survival? We compared lifespans of wild-type, outbred female flies that were continuously fed a diet containing sucrose concentration optimal for lifespan (5% sucrose, referred to as 1× sugar [1×S]; Bass et al., 2007) to that of their sisters, which were transiently fed an 8× excess of sugar (8×S) starting from day 2 of adulthood (Figure 1A). 8×S diet has both an increased caloric value and skewed protein-to-carbohydrate ratio. We limited treatment time to 3 weeks (a third of median life expectancy) to circumvent premature mortality that results from consuming this diet long term (Al Saud et al., 2015; Skorupa et al., 2008), thus avoiding potential bias arising from selection of hardy individuals. Fewer than 10% of experimental flies died during treatment (Figure 1B).

To evaluate the persistent, long-lasting effects of 8×S diet, we examined survival when all the flies were back on 1×S food. We found that the median lifespan of flies that had been fed 8×S for 3 weeks was reduced (7%; Figure 1B). The effect could not be attributed to changes in feeding after the exposure to 8×S food, because no differences in feeding or body mass were observed after 1 week of recovery on 1×S (Figures S1A and S1B). Interestingly, excess sugar did not impact survival immediately after treatment but created a vulnerability to the effects of age (Figure 1B). Statistical modeling using Cox proportional hazards (CPHs) confirmed that the time spent on 8×S before 23 days of age significantly increased the risk of death after 23 days ($p < 2 \times 10^{-16}$; Table S1). We noticed that different treatment groups had different median but similar maximum lifespans, prompting us to examine whether the effect of 8×S decayed with time. The increase in risk of death decayed with time ($p < 2 \times 10^{-16}$; Table S1), implying either that the flies had a heterogeneous response to sugar, potentially due to the genetic variation in the outbred population, or that the effect of 8×S feeding was slowly erased.

To ensure that the effect of 8×S feeding was substantially long term and to better estimate its magnitude, we examined the demography of survival in middle and old age, between 40 and 80 days, by analyzing over 1,000 deaths. Having been exposed to 8×S in early adulthood significantly increased relative risk of death in both mid-life (40–60 days interval) and late life (61–80 days interval), with 3 weeks on 8×S increasing the relative

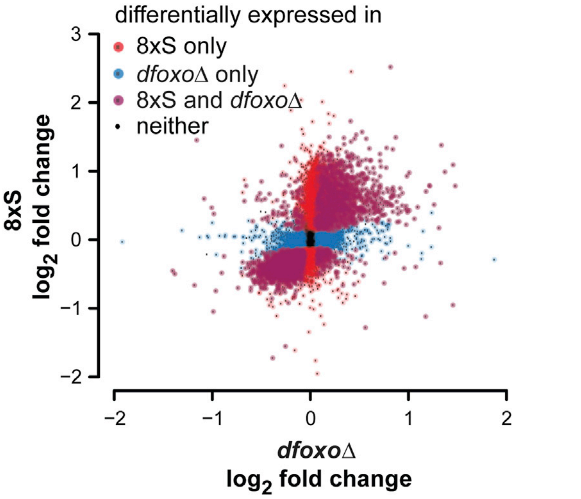

Figure 2. Transcriptional Response to Sugar Implicates dFOXO

The transcriptional response to 8×S is plotted against the response induced by deletion of *dfoxo*. Genes with significant differential expression (FDR = 10%) in either or both conditions are indicated. Note that only the genes present in both datasets are included. See also Data S1.

risk by ~50% (Figure S1C). Notably, the magnitude of this effect was comparable to the reported 91% increase in the relative, all-cause mortality risk in middle-aged and older humans who were obese as young adults independently of their BMI later in life (Hirko et al., 2015). Hence, excess sugar consumption in early adulthood has long-term detrimental effects in the fruit fly.

Such detrimental effects could be due to either programming of fly physiology or accumulation of irreparable molecular damage. We reasoned that, if damage caused the long-term effects of sugar-rich diets in the first third of life, then feeding on a healthy diet (1×S) in the same period, before any major mortality occurs, should be beneficial regardless of the subsequent diet (Figure 1C). We found no evidence of improved survival after feeding on 1×S (Figures 1D, S1D, and S1E). This indicates that, rather than cause irreparable damage, the relative amount of sugar consumed in a fly's early adulthood triggers a lasting physiological change or program, which can be detrimental in later life. In mammals, such persistent effects of nutrition are referred to as nutritional programming (Lucas, 1998). Overall, our data are consistent with nutritional programming of lifespan by relative sugar levels encountered in early adulthood in *Drosophila*.

Transcriptional Response to Sugar Implicates dFOXO

Next, we sought a regulatory mechanism whereby the 8×S diet programs lifespan. A proposed mechanism for mediating nutritional programming is the regulation of gene expression (Barnes and Ozanne, 2011; Niculescu and Lupu, 2011). We chose to identify TFs responsive to 8×S as candidates for mediating the long-term effects of this diet. To explore the transcriptional signature of sugar in *Drosophila*, we used RNA sequencing (RNA-seq) to interrogate whole-body transcriptomes of females fed 8×S or 1×S diet for 1 week, because any long-lasting programs must be a consequence of the changes occurring during exposure to the diet. A total of 6,435 genes were differentially expressed on 8×S (10% false discovery rate [FDR]; Figure 2; all gene lists are given in Data S1). Interestingly, we found that the promoters of genes repressed by 8×S were enriched for fork-head-like binding motifs (Data S1). FOXO TFs are evolutionarily conserved longevity determinants: activation of FOXO orthologs can extend lifespan in budding yeast, worms, and flies, and human *Foxo3* is one of only two genes consistently associated with longevity (Giannakou et al., 2004; Hwangbo et al., 2004; Kenyon et al., 1993; Morris et al., 2015; Postnikoff et al., 2012).

To explore whether the sole fly FOXO ortholog, *dfoxo*, may be involved in the transcriptional response to 8×S, we compared the list of sugar-responsive genes to the previously published set of genes differentially expressed in *dfoxo⊿* females (Alic et al., 2011). We found that the expression of 60% of sugar-responsive genes was also altered in *dfoxo⊿* flies, representing a highly significant overlap ($p = 9.4 \times 10^{-181}$; Figure 2). The transcriptional changes tended to be in the same direction between the two treatments (Figure 2), suggesting that dFOXO is inhibited by high sugar. We used Gene Ontology (GO) enrichment analysis on the genes regulated by both 8×S and *dfoxo* to predict the functional consequences of this inhibition. We found an enrichment of genes encoding chromatin modifiers, such as a range of chromatin/nucleosome remodelers and histone-modifying enzymes (Data S1), implicating epigenetic mechanisms in the long-term effects of sugar downstream of *dfoxo*. Overall, the transcriptional response pointed toward dFOXO as a candidate mechanistic mediator of nutritional programming by a sugar-rich diet in *Drosophila*, which we pursued further.

dFOXO Is Inhibited on Sugar-Rich Diet and Required for Transcriptional Changes that Mark Dietary History

We next examined whether dFOXO is regulated on 8×S diet. dFOXO is inhibited by the signaling cascade initiated by *Drosophila* insulin-like peptides (DILPs) (Teleman, 2009). We found that one of these, *dilp6*, was induced after 1 week of feeding on the 8×S diet ($p < 0.05$; Figure 3A). Such an increase in insulin/insulin-like growth factor (IGF) signaling is expected to result in phosphorylation and inhibition of dFOXO (Brunet et al., 1999; Alic et al., 2011). Indeed, we found that dFOXO phosphorylation was increased on the 8×S diet ($p < 0.05$; Figure 3B). To confirm that this phosphorylation impacts dFOXO's transcriptional activity, we selected several genes identified as responsive to both the 8×S diet and *dfoxo* deletion and examined whether their transcript levels were modulated by 8×S in a *dfoxo*-dependent manner. We focused on genes encoding epigenetic regulators due to their likely relevance to the legacy of the 8×S diet. The transcript levels of *Acf*, encoding a chromatin assembly factor subunit, *D12*, a subunit of the ATAC histone acetyltransferase complex, *egg*, a histone methyltransferase, *HDAC1*, a histone deacetylase, and *Hmt4-20*, a histone methyltransferase, were all reduced in *dfoxo⊿* flies ($p < 0.005$) and in the wild-type females fed 8×S diet ($p < 0.05$; Figure 3C). Importantly, 8×S did not repress these transcripts in *dfoxo⊿* flies ($p = 6 \times 10^{-4}$ for genotype by diet interaction; Figure 3C), confirming diet-induced changes as mediated by dFOXO inhibition. Hence, sugar-rich diet induces *dilp6* and inhibits dFOXO to repress dFOXO target genes, including epigenetic modifiers.

OPEN
ACCESS
CellPress

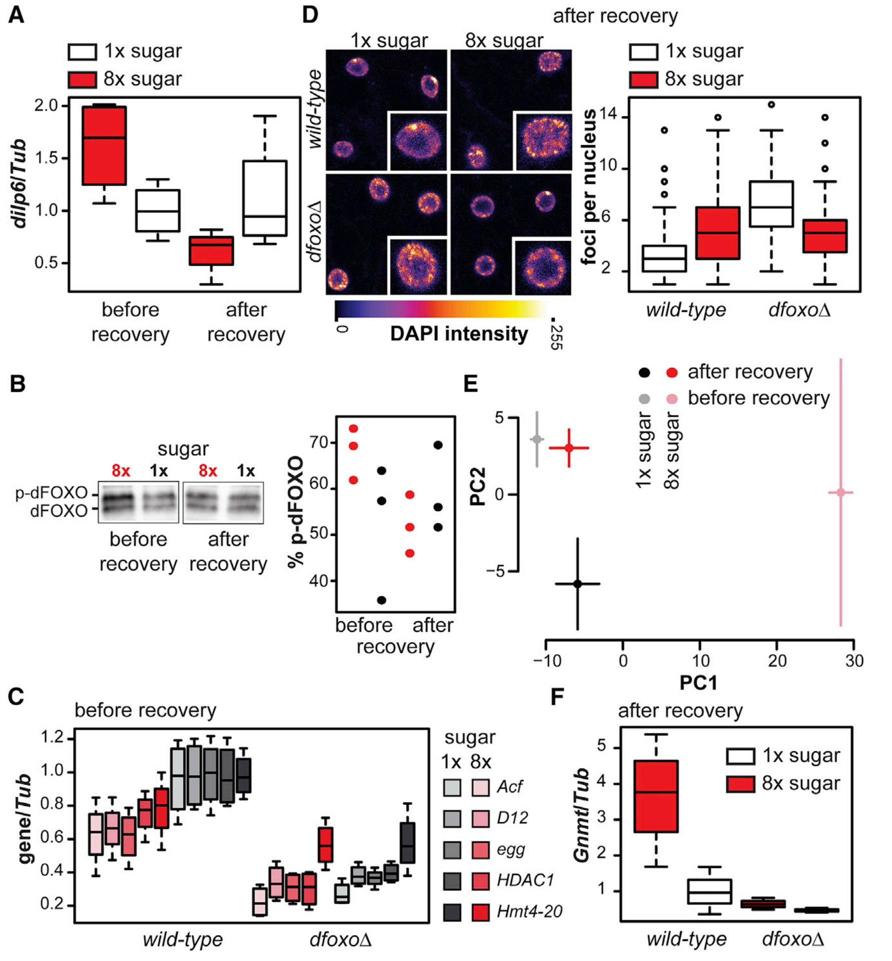

Figure 3. dFOXO Activity Is Regulated in Response to a Sugar-Rich Diet and Is Required for Transcriptional Memory of Past Diet

(A) *dilp6* transcript levels in wild-type females kept on 8×S or 1×S for 1 week (before recovery) and then allowed to recover on 1×S for 1 week (after recovery). Data were scaled to 1×S before recovery and analyzed with a linear model: the interaction between diet and time was significant ($p = 0.01$; $n = 4$). *dilp6* was induced significantly before ($p < 0.05$; two-tailed t test), but not after, recovery.

(B) Phosphorylated and unphosphorylated forms of dFOXO were separated by SDS-PAGE in whole-fly protein extracts obtained from females kept on 8×S or 1×S for 3 weeks and recovered on 1×S for 1 week. Quantifications from three repeats are shown to the right. The data were analyzed with a mixed-effects linear model, with repeat as a random effect: there was no significant effect of diet or time, but their interaction was significant ($p = 0.024$). 8×S was significantly different from 1×S before (t test; $p < 0.05$), but not after, recovery.

(C) Transcript levels of five genes encoding chromatin modifiers in wild-type and *dfoxoΔ* females after 1 week feeding on 1×S or 8×S. Data were scaled to wild-type levels on 1×S and analyzed with a linear model: the effects of transcript, diet, and genotype were significant ($p < 0.005$; $n = 4$). Interactions between transcript and other covariates were not significant, and genotype interacted with diet ($p = 6 \times 10^{-4}$): diet had a significant effect in the wild-type ($p < 0.05$; t test), but not *dfoxoΔ*.

(D) Distribution of DAPI staining in nuclei of abdominal fat body cells after recovery from 8×S in wild-type and *dfoxoΔ* females. DAPI intensity is false-colored for clarity. The quantification of the number of foci per nucleus is given along the representative images. Single cell inserts are ~10 × 10 μm. Generalized linear model with a Poisson distribution revealed a significant effect of genotype and significant interaction of genotype with diet ($n = 50$–90 nuclei from four or five animals; $p < 10^{-4}$).

(E) PCA analysis of transcriptomes of flies exposed to 8×S or 1×S for 1 week (before recovery; see Figure 2) or those allowed to recover for 1 week on 1×S (after recovery). Points show means ± SEs. PC1 and 2 collectively account for ~60% of total variance in the dataset.

(F) *Gnmt* transcript levels in wild-type or *dfoxoΔ* females kept on 8×S or 1×S for 1 week and then allowed to recover on 1×S for 1 week. Data are scaled to wild-type on 1×S and analyzed with a liner model: interaction between genotype and diet was significant ($p = 0.03$; $n = 3$ or 4), with *Gnmt* induced in the wild-type after 8×S feeding ($p < 0.05$; t test), but not in *dfoxoΔ*.

See also Figure S2.

The induction of *dilp6*, phosphorylation of dFOXO, and repression of its targets did not persist after recovery on 1×S diet (Figures 3A, 3B, and S2A). However, the transient repression of dFOXO may have long-term consequences through the observed, *dfoxo*-dependent regulation of epigenetic modifiers. To find evidence of long-term changes to chromatin, we examined nuclear DNA distribution in the fat body, an adipose-like organ in which dFOXO activity extends lifespan (Giannakou et al., 2004; Hwangbo et al., 2004). Nuclear DNA distribution is indicative of global chromatin arrangements (Fedorova and Zink, 2009; Tian et al., 2016). The frequency of DNA foci staining brightly with DAPI was increased after recovery from 8×S in the wild-type, but not *dfoxoΔ*, flies, which already displayed high levels on 1×S (Figure 3D), consistent with dfoxo-dependent resetting of chromatin states by 8×S. Such changes in chromatin states would be expected to result in persistent transcriptional changes. To examine whether any such changes exist, we performed RNA-seq on flies that consumed 8×S or 1×S food for 1 week and then recovered on 1×S for 1 week. This experiment was performed at the same time as analysis of transcriptional changes during 8×S feeding described above, allowing us to compare expression both before and after recovery. For an unbiased assessment of global transcriptional changes, we performed principal-component analysis (PCA) on all samples. PCA suggested an overall difference in the transcriptomes of flies that had recovered from the 8×S diet and their sisters, which were continuously kept on 1×S, indicating there is a long-term transcriptional program set up by 8×S feeding (Figure 3E). This program was distinct from the response observed before recovery both globally (Figure 3E) and at the level of differential expression

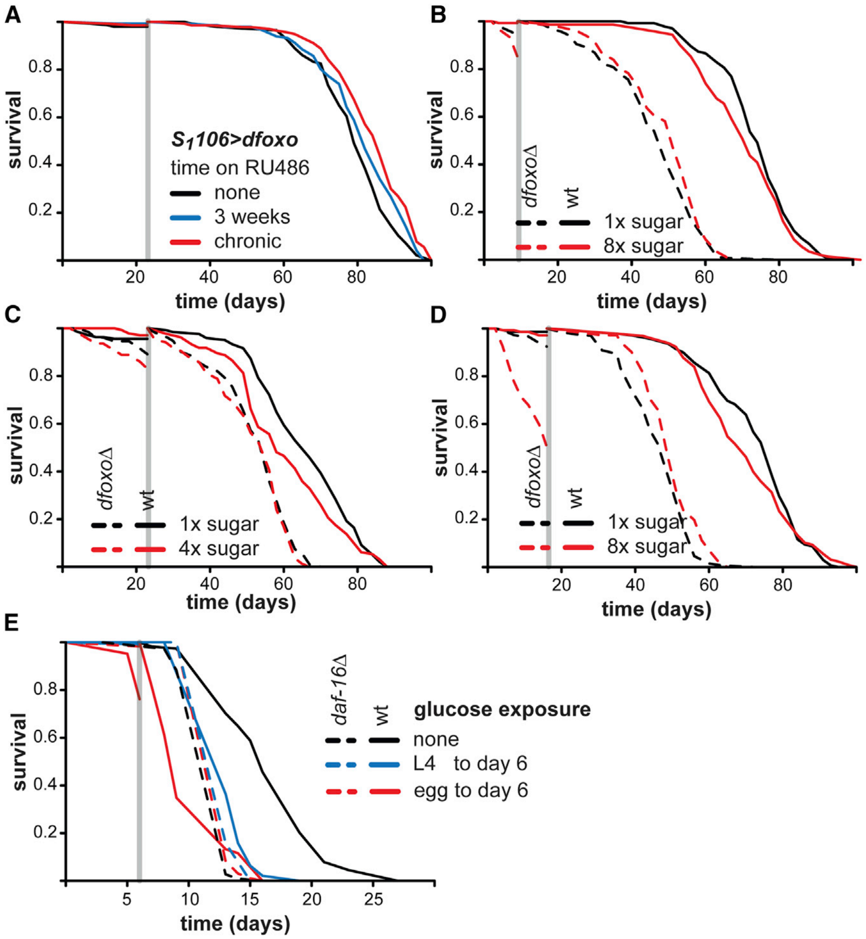

Figure 4. *dfoxo* and *daf-16* Are Required for Late-Life Detrimental Effects of Early-Life Diet High in Sugar

(A) *dfoxo* was induced in the gut/fat body of adult females with the S_1*106* driver from day 2 of adulthood, either chronically or for 3 weeks. Both acute and chronic *dfoxo* overexpression extended lifespan after day 23 ($p < 0.05$; log rank test).

(B–D) Survival of wild-type or *dfoxo⊿* females after feeding on 8×S for 1 week (B), 4×S for 3 weeks (C), or 8×S for 2 weeks (D) compared to those continuously kept on 1×S.

(E) Survival of wild-type or *daf-16* worms after treatment with glucose starting from either embryo (egg) or L4 and lasting up to day 6 of adulthood.

In all panels, the gray vertical bar indicates the time of switch, when survival was reset to 1. See also Tables S2–S5.

of individual genes (Figure S2B; Data S1), indicating it is not simply a carryover of the changes occurring before recovery. Hence, feeding with 8×S appears to place a novel, historic signature upon the fly's transcriptome.

Genes differentially expressed after recovery were not enriched for forkhead-like motifs in their promoters or for genes differentially expressed in *dfoxo* nulls, consistent with cessation of dFOXO repression after recovery. To address whether *dfoxo* is required for the transcriptional legacy of the 8×S diet, we profiled the mRNA levels of the gene encoding glycine N-methyltransferase (*Gnmt*), chosen because it was the most highly induced gene in response to 8×S after recovery (Figure S2B). We confirmed that *Gnmt* mRNA levels were significantly induced by prior exposure to 8×S diet after 1 week of recovery in wild-type flies ($p < 0.05$; Figure 3F). *Gnmt* could simply be responding to the long-term changes in the levels of its substrate, S-adenosyl-methionine (SAM). However, we found that, even though SAM levels increased on 8×S diet, the increase did not persist and could not account for increased *Gnmt* levels after recovery (Figure S2C). Hence, *Gnmt* induction is a transcriptional marker of the history of high-sugar feeding independent of current SAM levels. Importantly, *Gnmt* induction was not observed in *dfoxo⊿* females ($p = 0.03$ for genotype by sugar interaction; Figure 3F), revealing that *dfoxo* is required for this transcriptional memory of past sugar consumption. Overall, our data imply that a high-sugar diet reversibly inhibits dFOXO to cause short- and long-term transcriptional changes, perhaps accounting for the programming of lifespan observed.

dfoxo/*daf-16* Are Required for Programming of Lifespan by Early-Life Sugar-Rich Diet

Our data suggested that altered dFOXO activity in early life can influence subsequent survival. Indeed, 3-week induction of *dfoxo* in the gut and fat body of adult females using the tissue-specific, inducible, S_1*106* driver was sufficient to extend their subsequent lifespan ($p < 0.05$; Figure 4A; Giannakou et al., 2007). Importantly, dFOXO levels have been shown to revert back to normal after induction ceases (Giannakou et al., 2007). Whereas the lifespan effect of transient induction was less than achieved by chronic induction (Figure 4A), it confirmed that dFOXO activity in early life can have long-term consequences.

Is *dfoxo* required for the long-term, detrimental effect of a sugar-rich diet? We tested this in three independent experiments. *dfoxo⊿* females are short lived, both on 1×S and 8×S (Al Saud et al., 2015). To avoid bias that could result from a strong selection on the *dfoxo⊿* population, we limited the exposure to 8×S in early adulthood to only 1 week in the first experiment (Figure 4B). In the second, we limited the dose of sugar to 4×S for 3 weeks (Figure 4C). Finally, we tested whether 2 weeks on 8×S, which resulted in nearly 50% of the *dfoxo⊿* population dying during treatment, could elicit a response in the surviving *dfoxo⊿* flies (Figure 4D). We assessed survival after treatment to isolate the effects of dietary history. Transient feeding with sugar-rich diets and the deletion of *dfoxo* both had a significant, detrimental effect on subsequent survival ($p < 2 \times 10^{-16}$ in all experiments; Tables S2–S4). Importantly, historical exposure to sugar-rich diets did not further shorten the lifespan of *dfoxo⊿* females in any experiment ($p < 0.05$ for interaction between

diet and genotype; Figures 4B–4D; Tables S2–S4), revealing that *dfoxo* is required for the effects of dietary history on survival. This implies that a high-sugar diet in early adulthood acts through *dfoxo* to program lifespan.

We sought to establish whether the role of dFOXO in nutritional programming of lifespan was conserved in its worm ortholog, DAF-16. Chronic exposure to glucose reduces worm lifespan by inhibiting DAF-16 (Lee et al., 2009; Schulz et al., 2007), but the effects of transient exposure have not been examined. Presence of additional glucose in the media throughout development or during the first 6 days of adulthood (a third of median life expectancy) reduced the subsequent survival in wild-type worms ($p < 2 \times 10^{-16}$; Figure 4E; Table S5), revealing a lasting detrimental effect equivalent to that in the fly. Importantly, the survival of the *daf-16* worms was not sensitive to glucose in early life (Figure 4E; $p < 2 \times 10^{-16}$ for interaction between diet and genotype; Table S5). Hence, the role of FOXO factors in mediating nutritional programming of lifespan is evolutionarily conserved between flies and worms, making it likely that they play an equivalent role in nutritional programming in mammals.

DISCUSSION

Epidemiological and other data have provided extensive evidence that nutrition in early life can have lasting consequences for aging and age-related disease in mammals, including humans (Alfaradhi and Ozanne, 2011; Barnes and Ozanne, 2011). However, to date, it has been unclear whether any of the several evolutionarily conserved, longevity-assurance mechanisms that have been discovered in simpler animal models (Gems and Partridge, 2013) connect early nutrition to health and survival in later life. Our study strongly implicates FOXO factors as this missing mechanistic link between early-life nutrition and longevity. The strong evolutionary conservation of FOXO function makes it highly likely that FOXO factors play a role in some aspect of nutritional programming in mammals.

The role of dFOXO in mediating the long-term effects of a sugar-rich diet in *Drosophila* is surprisingly specific. dFOXO is not required for the lifespan benefits of a chronic reduction in protein intake, even though its activity can modulate the response (Giannakou et al., 2008). Similarly, the survival of *dfoxo*-null flies is reduced by chronic feeding with a sugar-rich diet to the same extent as the wild-type's (Al Saud et al., 2015). These differences in the role of dFOXO in response to chronic or acute dietary regimes, or different dietary components, may arise from the complex interactions between nutrition and insulin/IGF-like signaling: each DILP is expressed in a unique tissue pattern, acts in an endocrine and/or paracrine manner, and responds distinctly to the relative amounts of protein and carbohydrate present in the diet (Post and Tatar, 2016). This, in turn, may specify the tissues in which dFOXO is inhibited by specific diets and the nature of dFOXO targets affected.

In aging studies, the principal focus is on discovering and understanding mechanisms whereby lifespan can be extended and health maintained in later life. Several instances already exist where a transient intervention, be it during development or in adulthood, can have prolonged beneficial consequences (Bitto et al., 2016; Dillin et al., 2002; Schulz et al., 2007). For example, mild impairment of mitochondrial function during worm development can extend adult lifespan (Dillin et al., 2002), and these long-term effects are mediated by epigenetic changes (Merkwirth et al., 2016; Tian et al., 2016). We have investigated how lifespan is curtailed, rather than extended, by unhealthy nutrition in early adulthood. Similar to transient, lifespan-extending interventions, it is likely that the long-term, detrimental effects of diet-induced FOXO inhibition are also due to persistent epigenetic modifications. Indeed, DAF-16 engages the SWI/SNF chromatin-remodeling complex to increase worm lifespan (Riedel et al., 2013). Our gene expression data show that, in *Drosophila*, a sugar-rich diet represses dFOXO to drive changes in expression of a number of epigenetic modifiers, which is likely to have substantial consequences for the epigenome. Elucidating these *dfoxo*-dependent epigenetic changes and how they could be reversed may form the basis of future treatments to remedy the cost of past diets.

EXPERIMENTAL PROCEDURES

Fly Husbandry, Food, Feeding, and Lifespan Assays

Outbred, Dahomey fly population carrying the w^{1118} mutation was used in all experiments. *D. melanogaster* diet contained 10% yeast, 1.5% agar with 40% (8×S) or 5% (1×S) sucrose (all w/v). When required, RU486 (200 μM) was included in the food. *C. elegans* were reared as per Brenner (1974) and worms exposed to 2% glucose in NGM plates from embryo or from L4 stage. RNA was isolated with Trizol for qPCR and RNA sequencing. Protein samples were extracted in TCA and dFOXO phosphorylation assessed as described (Alic et al., 2011; Giannakou et al., 2007). Transcriptomes were analyzed by aligning reads to *Drosophila* genome dm6 in Tophat2, enumerating reads with HTSeq, and model fitting using DESeq2. TF-binding motif enrichment was analyzed using iRegulon. Survival was analyzed in R and JMP. See Supplemental Experimental Procedures for full details of animal husbandry, molecular, and data analysis.

ACCESSION NUMBERS

The accession number for the RNA-seq data reported in this paper is ArrayExpress: E-MTAB-4766.

SUPPLEMENTAL INFORMATION

Supplemental Information includes Supplemental Experimental Procedures, two figures, five tables, and one data file and can be found with this article online at http://dx.doi.org/10.1016/j.celrep.2016.12.029.

AUTHOR CONTRIBUTIONS

N.A. devised the study; A.J.D., M.E., C.E.F., and A.C.S. performed experiments; A.J.D., M.E., C.E.F., A.C.S., and N.A. analyzed the data; M.D.W.P., D.G., and N.A. supervised the study; and A.J.D., M.E., M.D.W.P., D.G., and N.A. wrote the manuscript.

ACKNOWLEDGMENTS

We acknowledge funding from the Biotechnology and Biological Sciences Research Council (to N.A.; BB/M029093/1), Medical Research Council (to N.A.; MR/L018802/1), Royal Society (to N.A.: RG140694; to M.D.W.P.: UF100158 and RG110303), Wellcome Trust (to D.G.; WT098565/Z/12/Z), and Australian Research Council (to M.D.W.P.; FT150100237). We thank D. Ivanov for help with RNA sequencing analysis; C. Slack and L. Partridge for reagents; and Y. Feseha, K. Shalfrooshan, and Z. Alqabandi for technical assistance.

Received: May 19, 2016
Revised: October 21, 2016
Accepted: December 8, 2016
Published: January 10, 2017

REFERENCES

Al Saud, S.N., Summerfield, A.C., and Alic, N. (2015). Ablation of insulin-producing cells prevents obesity but not premature mortality caused by a high-sugar diet in *Drosophila*. Proc. Biol. Sci. *282*, 20141720.

Alfaradhi, M.Z., and Ozanne, S.E. (2011). Developmental programming in response to maternal overnutrition. Front. Genet. *2*, 27.

Alic, N., Andrews, T.D., Giannakou, M.E., Papatheodorou, I., Slack, C., Hoddinott, M.P., Cochemé, H.M., Schuster, E.F., Thornton, J.M., and Partridge, L. (2011). Genome-wide dFOXO targets and topology of the transcriptomic response to stress and insulin signalling. Mol. Syst. Biol. *7*, 502.

Barnes, S., and Ozanne, S. (2011). The role of epigenetics in developmental programming. Practical Diabetes *28*, 245–246.

Bass, T.M., Grandison, R.C., Wong, R., Martinez, P., Partridge, L., and Piper, M.D. (2007). Optimization of dietary restriction protocols in Drosophila. J. Gerontol. A Biol. Sci. Med. Sci. *62*, 1071–1081.

Bitto, A., Ito, T.K., Pineda, V.V., LeTexier, N.J., Huang, H.Z., Sutlief, E., Tung, H., Vizzini, N., Chen, B., Smith, K., et al. (2016). Transient rapamycin treatment can increase lifespan and healthspan in middle-aged mice. eLife *5*, e16351.

Brenner, S. (1974). The genetics of Caenorhabditis elegans. Genetics *77*, 71–94.

Brunet, A., Bonni, A., Zigmond, M.J., Lin, M.Z., Juo, P., Hu, L.S., Anderson, M.J., Arden, K.C., Blenis, J., and Greenberg, M.E. (1999). Akt promotes cell survival by phosphorylating and inhibiting a Forkhead transcription factor. Cell *96*, 857–868.

Buescher, J.L., Musselman, L.P., Wilson, C.A., Lang, T., Keleher, M., Baranski, T.J., and Duncan, J.G. (2013). Evidence for transgenerational metabolic programming in Drosophila. Dis. Model. Mech. *6*, 1123–1132.

Christensen, K., Doblhammer, G., Rau, R., and Vaupel, J.W. (2009). Ageing populations: the challenges ahead. Lancet *374*, 1196–1208.

Dearden, L., and Ozanne, S.E. (2015). Early life origins of metabolic disease: developmental programming of hypothalamic pathways controlling energy homeostasis. Front. Neuroendocrinol. *39*, 3–16.

Dillin, A., Hsu, A.L., Arantes-Oliveira, N., Lehrer-Graiwer, J., Hsin, H., Fraser, A.G., Kamath, R.S., Ahringer, J., and Kenyon, C. (2002). Rates of behavior and aging specified by mitochondrial function during development. Science *298*, 2398–2401.

Fedorova, E., and Zink, D. (2009). Nuclear genome organization: common themes and individual patterns. Curr. Opin. Genet. Dev. *19*, 166–171.

Fernandez-Twinn, D.S., Alfaradhi, M.Z., Martin-Gronert, M.S., Duque-Guimaraes, D.E., Piekarz, A., Ferland-McCollough, D., Bushell, M., and Ozanne, S.E. (2014). Downregulation of IRS-1 in adipose tissue of offspring of obese mice is programmed cell-autonomously through post-transcriptional mechanisms. Mol. Metab. *3*, 325–333.

Gems, D., and Partridge, L. (2013). Genetics of longevity in model organisms: debates and paradigm shifts. Annu. Rev. Physiol. *75*, 621–644.

Giannakou, M.E., Goss, M., Jünger, M.A., Hafen, E., Leevers, S.J., and Partridge, L. (2004). Long-lived Drosophila with overexpressed dFOXO in adult fat body. Science *305*, 361.

Giannakou, M.E., Goss, M., Jacobson, J., Vinti, G., Leevers, S.J., and Partridge, L. (2007). Dynamics of the action of dFOXO on adult mortality in Drosophila. Aging Cell *6*, 429–438.

Giannakou, M.E., Goss, M., and Partridge, L. (2008). Role of dFOXO in lifespan extension by dietary restriction in Drosophila melanogaster: not required, but its activity modulates the response. Aging Cell *7*, 187–198.

Gillman, M.W. (2005). Developmental origins of health and disease. N. Engl. J. Med. *353*, 1848–1850.

Hardikar, A.A., Satoor, S.N., Karandikar, M.S., Joglekar, M.V., Puranik, A.S., Wong, W., Kumar, S., Limaye, A., Bhat, D.S., Januszewski, A.S., et al. (2015). Multigenerational undernutrition increases susceptibility to obesity and diabetes that is not reversed after dietary recuperation. Cell Metab. *22*, 312–319.

Hirko, K.A., Kantor, E.D., Cohen, S.S., Blot, W.J., Stampfer, M.J., and Signorello, L.B. (2015). Body mass index in young adulthood, obesity trajectory, and premature mortality. Am. J. Epidemiol. *182*, 441–450.

Hwangbo, D.S., Gershman, B., Tu, M.P., Palmer, M., and Tatar, M. (2004). Drosophila dFOXO controls lifespan and regulates insulin signalling in brain and fat body. Nature *429*, 562–566.

Kenyon, C., Chang, J., Gensch, E., Rudner, A., and Tabtiang, R. (1993). A C. elegans mutant that lives twice as long as wild type. Nature *366*, 461–464.

Langley-Evans, S.C. (2006). Developmental programming of health and disease. Proc. Nutr. Soc. *65*, 97–105.

Lee, K.P., Simpson, S.J., Clissold, F.J., Brooks, R., Ballard, J.W., Taylor, P.W., Soran, N., and Raubenheimer, D. (2008). Lifespan and reproduction in Drosophila: new insights from nutritional geometry. Proc. Natl. Acad. Sci. USA *105*, 2498–2503.

Lee, S.J., Murphy, C.T., and Kenyon, C. (2009). Glucose shortens the life span of C. elegans by downregulating DAF-16/FOXO activity and aquaporin gene expression. Cell Metab. *10*, 379–391.

Lucas, A. (1998). Programming by early nutrition: an experimental approach. J. Nutr. *128* (*2, Suppl*), 401S–406S.

Lustig, R.H., Schmidt, L.A., and Brindis, C.D. (2012). Public health: the toxic truth about sugar. Nature *482*, 27–29.

Mair, W., Goymer, P., Pletcher, S.D., and Partridge, L. (2003). Demography of dietary restriction and death in Drosophila. Science *301*, 1731–1733.

Mair, W., Piper, M.D., and Partridge, L. (2005). Calories do not explain extension of life span by dietary restriction in Drosophila. PLoS Biol. *3*, e223.

Merkwirth, C., Jovaisaite, V., Durieux, J., Matilainen, O., Jordan, S.D., Quiros, P.M., Steffen, K.K., Williams, E.G., Mouchiroud, L., Tronnes, S.U., et al. (2016). Two conserved histone demethylases regulate mitochondrial stress-induced longevity. Cell *165*, 1209–1223.

Morris, B.J., Willcox, D.C., Donlon, T.A., and Willcox, B.J. (2015). FOXO3: a major gene for human longevity–a mini-review. Gerontology *61*, 515–525.

Niccoli, T., and Partridge, L. (2012). Ageing as a risk factor for disease. Curr. Biol. *22*, R741–R752.

Niculescu, M.D., and Lupu, D.S. (2011). Nutritional influence on epigenetics and effects on longevity. Curr. Opin. Clin. Nutr. Metab. Care *14*, 35–40.

Öst, A., Lempradl, A., Casas, E., Weigert, M., Tiko, T., Deniz, M., Pantano, L., Boenisch, U., Itskov, P.M., Stoeckius, M., et al. (2014). Paternal diet defines offspring chromatin state and intergenerational obesity. Cell *159*, 1352–1364.

Partridge, L., Green, A., and Fowler, K. (1987). Effects of egg production and of exposure to males on female survival in Drosophila melanogaster. J. Insect Physiol. *33*, 745–749.

Piper, M.D., Partridge, L., Raubenheimer, D., and Simpson, S.J. (2011). Dietary restriction and aging: a unifying perspective. Cell Metab. *14*, 154–160.

Post, S., and Tatar, M. (2016). Nutritional geometric profiles of insulin/IGF expression in Drosophila melanogaster. PLoS ONE *11*, e0155628.

Postnikoff, S.D., Malo, M.E., Wong, B., and Harkness, T.A. (2012). The yeast forkhead transcription factors fkh1 and fkh2 regulate lifespan and stress response together with the anaphase-promoting complex. PLoS Genet. *8*, e1002583.

Riedel, C.G., Dowen, R.H., Lourenco, G.F., Kirienko, N.V., Heimbucher, T., West, J.A., Bowman, S.K., Kingston, R.E., Dillin, A., Asara, J.M., and Ruvkun, G. (2013). DAF-16 employs the chromatin remodeller SWI/SNF to promote stress resistance and longevity. Nat. Cell Biol. *15*, 491–501.

Schulz, T.J., Zarse, K., Voigt, A., Urban, N., Birringer, M., and Ristow, M. (2007). Glucose restriction extends Caenorhabditis elegans life span by

inducing mitochondrial respiration and increasing oxidative stress. Cell Metab. *6*, 280–293.

Simpson, S.J., Le Couteur, D.G., and Raubenheimer, D. (2015). Putting the balance back in diet. Cell *161*, 18–23.

Skorupa, D.A., Dervisefendic, A., Zwiener, J., and Pletcher, S.D. (2008). Dietary composition specifies consumption, obesity, and lifespan in Drosophila melanogaster. Aging Cell *7*, 478–490.

Slagboom, P.E., Beekman, M., Passtoors, W.M., Deelen, J., Vaarhorst, A.A., Boer, J.M., van den Akker, E.B., van Heemst, D., de Craen, A.J., Maier, A.B., et al. (2011). Genomics of human longevity. Philos. Trans. R. Soc. Lond. B Biol. Sci. *366*, 35–42.

Tatar, M., Post, S., and Yu, K. (2014). Nutrient control of Drosophila longevity. Trends Endocrinol. Metab. *25*, 509–517.

Teleman, A.A. (2009). Molecular mechanisms of metabolic regulation by insulin in Drosophila. Biochem. J. *425*, 13–26.

Tian, Y., Garcia, G., Bian, Q., Steffen, K.K., Joe, L., Wolff, S., Meyer, B.J., and Dillin, A. (2016). Mitochondrial stress induces chromatin reorganization to promote longevity and UPR(mt). Cell *165*, 1197–1208.

van den Heuvel, J., Zandveld, J., Mulder, M., Brakefield, P.M., Kirkwood, T.B., Shanley, D.P., and Zwaan, B.J. (2014). The plastic fly: the effect of sustained fluctuations in adult food supply on life-history traits. J. Evol. Biol. *27*, 2322–2333.

Cell Reports, Volume *18*

Supplemental Information

Nutritional Programming of Lifespan by FOXO Inhibition on Sugar-Rich Diets

Adam J. Dobson, Marina Ezcurra, Charlotte E. Flanagan, Adam C. Summerfield, Matthew D.W. Piper, David Gems, and Nazif Alic

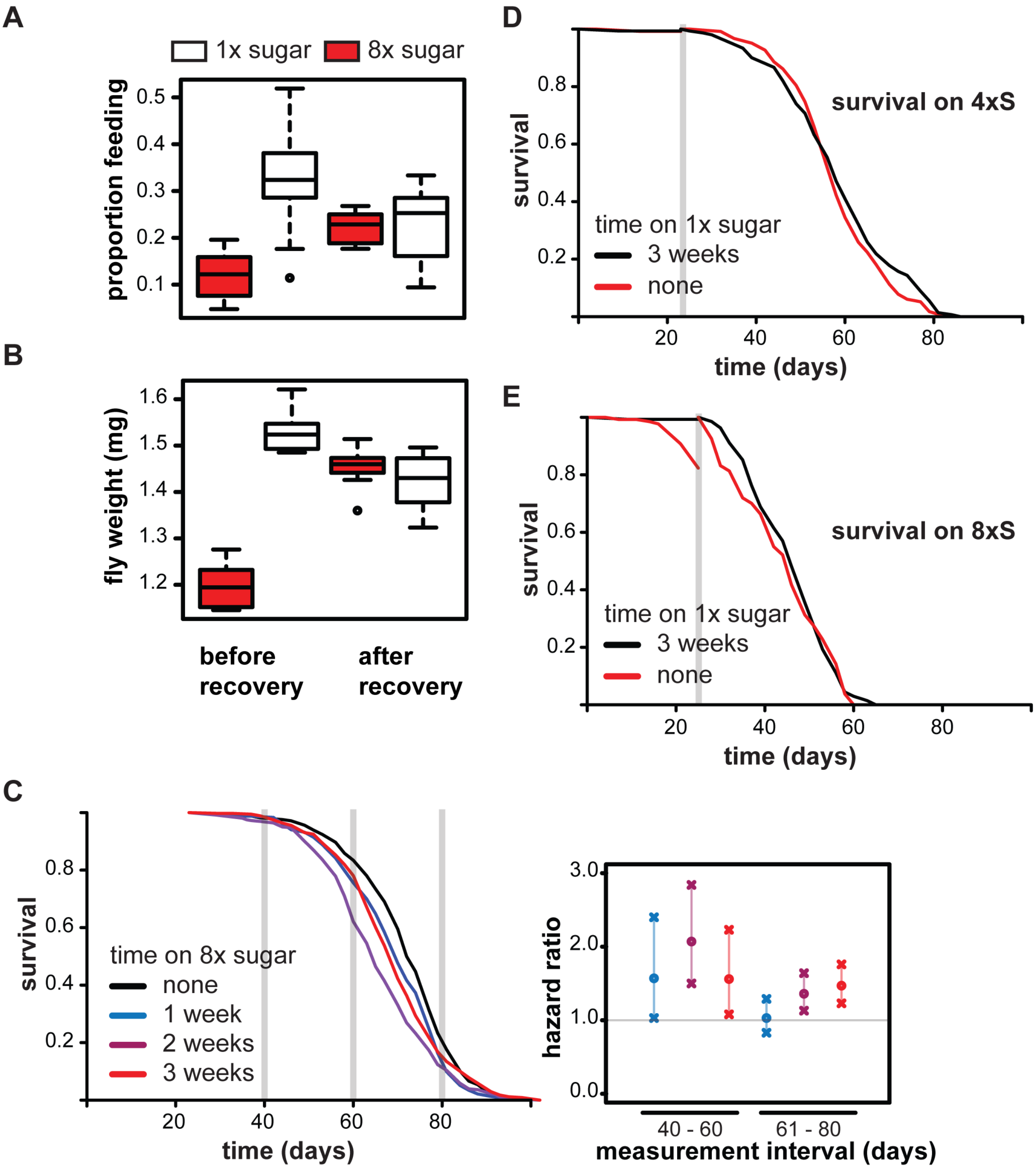

Figure S1. Related to Figure 1. Wild-type flies were fed 1xS or 8xS diet for a week and then allowed to recover on 1xS diet for a week. Proportion of flies feeding (**A**) and fly weight (**B**) were determined at the end of the first week (before recovery) and at the end of the second week (after recovery). Both feeding rate and body weight were significantly reduced by 8xS before but not after recovery (Body weight ANOVA diet * time interaction, p = $6x10^{-9}$; Proportion feeding GLM diet * time interaction, p=$8x10^{-4}$). **C** Survival curves pooled from multiple experiments (including those in the main figures) and the hazard ratios and 95% confidence intervals calculated from events in the indicated time periods. Total number of dead/censored flies: control: 651/22; 1 week treatment: 233/9 ; 2 weeks: 359/3; 3 weeks: 357/4. To calculate the hazard ratios a mixed-effect CPH model was fitted with experimental trial as a random effect. **D** and **E** – Survival on diets rich in sugar after 3 weeks (23 days for G) on 1xS compared to no exposure to 1xS. Time on 1xS did not significantly increase survival.

A

B

C

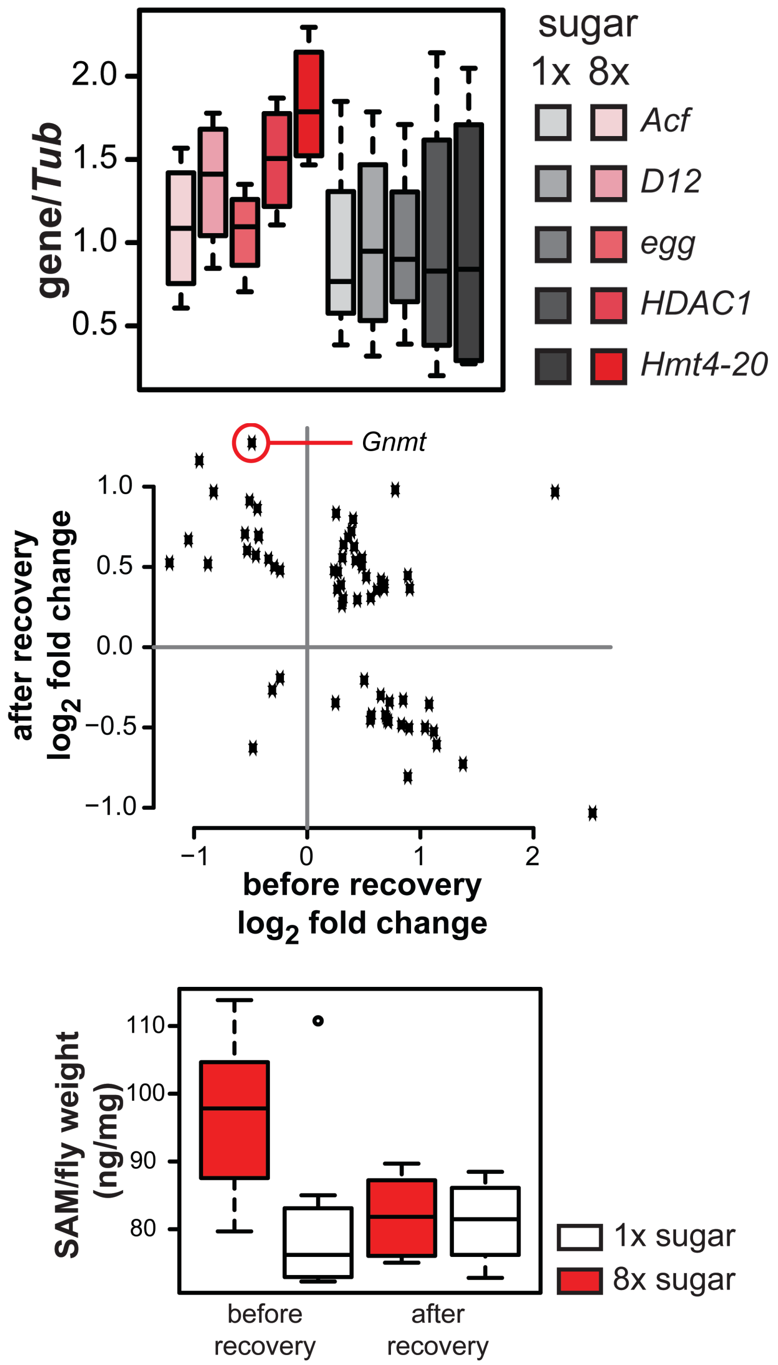

Figure S2. Related to Figure 3. A Expression of *Acf*, *D12*, *egg*, *HDAC1* and *Hmt4-20* after recovery in the wild-type. Data were scaled to 1xS and analysis with a liner model indicated a near-significant induction of all transcripts by 8xS after recovery (p=0.06). **B** Log_2 fold expression ~~change~~ induced by 8xS before and after recovery for the set of genes differentially expressed after recovery (10% FDR), with *Gnmt* indicated. **C** Levels of SAM before and after a week of recovery following from a week of treatment with indicated food. Data were analysed with a linear model, revealing a significant effect of sugar and time (p<0.05) and a significant interaction of the two (p=0.01) where SAM levels were elevated in response to 8xS before (p<0.05, t-test) but not after recovery.

Table S1. Related to Figure 1B. CPH analysis of survival data.

**coefficient***	**estimate****	**s.e.**	z	***p*-value**
time on 8x sugar	4.2	0.22	18.82	$<2x10^{-16}$
decay of sugar effect	-0.084	0.0046	-18.23	$<2x10^{-16}$

*Exposure to sugar was modeled as duration of exposure in weeks, the decay of the sugar effect was modeled as the interaction between the effect of sugar and time since exposure in days. **The estimate is the logarithm of the relative risk, where a positive value indicates an increase in relative risk of death. Total flies dead = 389, censored = 11.

Table S2. Related to Figure 4B. CPH analysis of survival data.

coefficient*	estimate**	s.e.	z	p-value
time on 8x sugar	9.1	0.55	17	$<2x10^{-16}$
decay of sugar effect	-0.13	$8.1x10^{-3}$	-16	$<2x10^{-16}$
foxoΔ	3.3	0.16	20	$<2x10^{-16}$
foxoΔ : time on 8x sugar	-2.1	0.66	-3.2	$1.4x10^{-3}$
foxoΔ : decay of sugar effect	-0.023	0.98	-1.8	0.077

*Time of exposure to 8x sugar was expressed in weeks, the decay of the sugar effect was modeled as the interaction between the effect of sugar and time since exposure in days, ":" indicates an interaction term.

**The estimate is the logarithm of the relative risk, where a positive value indicates an increase in relative risk of death. Total flies dead = 533, censored = 19.

Table S3. Related to Figure 4C. CPH analysis of survival data.

coefficient*	estimate**	s.e.	*z*	*p*-value
time on 4x sugar	2.6	0.15	17	$<2x10^{-16}$
decay of sugar effect	-0.053	$3.2x10^{-3}$	-16	$<2x10^{-16}$
foxoΔ	2.2	0.18	12	$<2x10^{-16}$
foxoΔ : time on 4x sugar	-0.4	0.17	-2.4	0.018
foxoΔ : decay of sugar effect	-0.011	$4.7x10^{-3}$	-2.4	0.019

*Time of exposure to 4x sugar was expressed in weeks, the decay of the sugar effect was modelled as the interaction between the effect of sugar and time since exposure in days, ":" indicates an interaction term.
**The estimate is the logarithm of the relative risk, where a positive value indicates an increase in relative risk of death.
Total flies dead = 434, censored = 26.

Table S4. Related to Figure 4D. CPH analysis of survival data.

**coefficient***	**estimate****	**s.e.**	***z***	***p*-value**
time on 8x sugar	4.4	0.28	15	$<2x10^{-16}$
decay of sugar effect	-0.07	$4.5x10^{-3}$	-15	$<2x10^{-16}$
foxoΔ	4	0.21	19	$<2x10^{-16}$
foxoΔ : time on 8x sugar	-1.4	0.41	-3.6	$3.5x10^{-4}$
foxoΔ : decay of sugar effect	-0.014	$9.7x10^{-3}$	-1.4	0.16

*Time of exposure to 8x sugar was expressed in weeks, the decay of the sugar effect was modelled as the interaction between the effect of sugar and time since exposure in days, ”:” indicates an interaction term.

**The estimate is the logarithm of the relative risk, where a positive value indicates an increase in relative risk of death. Total flies dead = 469, censored = 7.

Table S5. Related to Figure 4E. CPH analysis of survival data.

Start of treament*	coefficient**	estimate***	s.e.	*z*	*p*-value
embryo (egg)	glucose	2.2	0.2	11	$<2x10^{-16}$
	daf-16	1.9	0.18	11	$<2x10^{-16}$
	glucose : *daf-16*	-2.4	0.24	-10	$<2x10^{-16}$
L4	glucose	1.4	0.17	8.4	$<2x10^{-16}$
	daf-16	2.2	0.18	13	$<2x10^{-16}$
	glucose : *daf-16*	-1.8	0.16	-8.3	$<2x10^{-16}$

*Separate CPH models were fitted using the same control (no glucose) data.
**Glucose was fitted as categorical covariate, ":" indicates an interaction term.
***The estimate is the logarithm of the relative risk, where a positive value indicates an increase in relative risk of death.
Total worms dead/censored: from embryo (egg) = 379/23, from L4 = 417/20.

Extended experimental procedures

Fly husbandry, food, feeding and lifespan assays

dfoxoΔ^{94} mutant (Slack et al., 2011), *S*$_{1}$*106* and *UAS-dfoxo* (Giannakou et al., 2004) were backcrossed at least 6 times into the wild-type, outbred, Dahomey population carrying the w^{1118} mutation that was used in all experiments. The Dahomey stock was collected in 1970 in Dahomey (now Benin) and kept in population cages to maintain its lifespan and fecundity at levels similar to freshly caught stocks. The lines were maintained, and all experiments performed, at 25ºC with 60% humidity and 12h:12h light:dark cycle. Flies were maintained on food containing 10% yeast, 5% sucrose, 1.5% agar (all w/v; 1xS food) (Bass et al., 2007). Experimental flies were reared from embryo at standardised densities on 1xS food and females were sorted at random onto the appropriate food 48h after emergence and *ad libitum* mating. 8xS food contained 10% yeast, 40% sucrose, 1.5% agar. Lifespan experiments were performed as described previously (Al Saud et al., 2015) on cohorts of flies housed 10 females per vial. Feeding rate was measured by a proboscis extension assay, counting the number of flies feeding per instantaneous observation per vial, every 5 minutes for 2 hours between 1h and 3h into the light cycle, after overnight acclimatisation to the observation arena.

Worm husbandry, food and lifespans assays

Standard *C. elegans* culture was as previously described (Brenner, 1974). Strains were grown at 20°C on NGM plates seeded with *E. coli* OP50. N2 (wild type) and GR1307 *daf-16(mgDf50)* were used. For glucose experiments, 2% glucose was added to molten NGM before plate pouring. Survival assays were performed at 20°C on plates supplemented with 5-Fluoro-2'-deoxyuridine to 15µM to inhibit progeny growth.

RNA, qPCR, protein extractions, western blots, weight and SAM measurements, DAPI staining

10 females per sample were snap-frozen in liquid nitrogen. RNA was extracted with Trizol, converted to cDNA and qPCR performed using *dilp6* primers (Gronke et al., 2010) or the following primers:

Primers used for qPCR

Primer ID (gene, Fwd/Rev)	*Sequence*
Acf F	CGCGACTATGAACACTAC
Acf R	TCCTCGTAGGTGAGGTTC
D12 F	CTAATGCACACGGTGGTG
D12 R	ATCGCCTGTTTTGCTCTCAG
egg F	GCTGCGTGTCCCAAGACG

egg R	GGGCAAAGGCACGCATCTG
Gnmt F	GGAGGCGTCCTGCTTATC
Gnmt R	CCGTGTGACTCGTATTATAG
HDAC1 F	GTTTGTTACTACTACGACAGC
HDAC1 R	CGATAGAGCCCATAGTTG
Hmt4-20 F	CGGCTCCACGATCATATC
Hmt4-20 R	CCCGTTGCTCTTCCAGTG
Tubulin F	TGGGCCCGTCTGGACCACAA
Tubulin R	TCGCCGTCACCGGAGTCCAT

Proteins were extracted with TCA, separated by SDS-PAGE and transferred to nitrocellulose membranes and visualised using the antibody previously described (Alic et al., 2011; Giannakou et al., 2007). The slower migrating form has been shown to correspond to phosphorylated dFOXO (Alic et al., 2011).

For body weight and SAM assays, flies were CO_2 anaesthetised and weighed, then flash-frozen in liquid N_2. They were thawed on ice, homogenized with a micropestle in 75 µl ice-cold PBS, and SAM was assayed using the SAM Elisa Kit (Cell Biolabs STA-672) as per the manufacturer's instructions.

For DAPI staining, abdominal fat bodies, as associated with the cuticle, were dissected and fixed with 4% formaldehyde, washed and stained with DAPI. Confocal stacks were obtained on Zeiss LSM700. The number of bright spots per nucleus was determined in Volocity (PerkinElmer).

RNA sequencing and analysis

RNA sequencing (paired-end, 75bp per read) of poly-A RNA was performed by Glasgow Polyomics. The raw data have been submitted to Array Express (E-MTAB-4766). The reads were assessed for quality using FastQC, and aligned to Berkeley Drosophila Genome Project assembly release 6, where genes with exon overlap of >50% were combined into a pseudo-feature, and non-chromosomal regions excluded, using TopHat2. After converting BAM files to SAM files using SAMTOOLS, reads were counted using HTSeq (Anders et al., 2013). Four biological repeats were sequenced per treatment (one sample from the 8xS diet and its correspondent from the 1xS diet, both before recovery, were removed from subsequent analysis following preliminary assessment with PCA). Genes were filtered from differential expression analysis if ≤1 read was detected across all samples. Differential expression was analysed in R using a generalized linear model (GLM) in DESeq2 (Anders and Huber, 2010) using a beta prior. The GLM included each dietary condition (1 week of

exposure to 8xS/1xS, with/without 1 week of subsequent recovery on 1xS) as an unordered factor, and biological replicate as a cofactor. P values were adjusted using independent hypothesis weighting (Ignatiadis et al., 2016) with a significance threshold (alpha) of 0.1. Fold-changes in expression were calculated by the internal DESeq functions. *dfoxo* differential expression was as determined by (Alic et al., 2011) but at an FDR threshold of 0.1 to match the present study. Overlap between the sugar-responsive and *dfoxo*-responsive gene sets was determined by a hypergeometric test using the phyper() function in R. Principal components analysis was performed using the prcomp() function in R, on variance-stabilised reads (whole transcriptome) produced by DESeq2. GO enrichment was assessed with topGO (Alexa A and Rahnenfuhrer J (2016). topGO: Enrichment Analysis for Gene Ontology. R package version 2.24.0.) using a custom GO annotation accounting for pseudofeatures in the gtf file. Enrichment of GO terms was performed using the "weight01" algorithm to account for GO term structure, and applying Fisher's test for enrichment. Enrichment of transcription factor-binding motifs was analysed with iRegulon (Janky et al., 2014), using default parameters, and excluding features which are not annotated to bind known TFs. ~~Raw data have been deposited in ArrayExpress under the accession number: E-MTAB-4766.~~ The counts table, gene lists, outputs of GO and TF-binding site analysis are given in the **Supplemental Data**.

Statistical analysis

Survival data was analysed with Cox Proportional Hazards (CPH) in R using the "survival" package (Terry Therneau, http://CRAN.R-project.org/package=survival). Time-dependent covariates were dealt with as recommended by (Fox, 2008): the decay of the sugar effect was modelled as an interaction between the effect of sugar and time since end of treatment (time on 8x (weeks) : time since end of exposure (days)) so that the models described in supplemental tables took the form: Survival ~ time on 8x (weeks) + genotype + decay of sugar effect + genotype : time on 8x (weeks) + genotype : decay of sugar effect, where ":" specifies an interaction. For assessment of survival in specified intervals, all events occurring after the interval were included as censors and effects estimated using CHP: Survival ~ time on 8x (categorical) + experimental trial (random effect). qPCR data were scaled and analysed with a linear model in JMP (version 11) software (SAS Institute), followed by planned, pair-wise t-tests. Similarly, analysis of western blot quantification was performed with a mixed-effect linear model. Number of DAPI foci per nucleus was analysed with a generalised linear model with Poisson distribution in JMP. Log-rank test was performed in JMP. Details of statistical tests are given in figure and table captions.

Supplemental References

Al Saud, S., Summerfield, A., and Alic, N. (2015). Ablation of insulin-producing cells prevents obesity but not premature mortality caused by a high-sugar diet in *Drosophila*. Proc R Soc B *282*, 20141720.

Alic, N., Andrews, T.D., Giannakou, M.E., Papatheodorou, I., Slack, C., Hoddinott, M.P., Cocheme, H.M., Schuster, E.F., Thornton, J.M., and Partridge, L. (2011). Genome-wide dFOXO targets and topology of the transcriptomic response to stress and insulin signalling. Molecular Systems Biology *7*.

Anders, S., and Huber, W. (2010). Differential expression analysis for sequence count data. Genome Biol *11*, R106.

Anders, S., McCarthy, D.J., Chen, Y., Okoniewski, M., Smyth, G.K., Huber, W., and Robinson, M.D. (2013). Count-based differential expression analysis of RNA sequencing data using R and Bioconductor. Nat Protoc *8*, 1765-1786.

Bass, T.M., Grandison, R.C., Wong, R., Martinez, P., Partridge, L., and Piper, M.D. (2007). Optimization of dietary restriction protocols in Drosophila. J Gerontol A Biol Sci Med Sci *62*, 1071-1081.

Brenner, S. (1974). The genetics of Caenorhabditis elegans. Genetics *77*, 71-94.

Fox, J. (2008). Cox proportional-hazards regression for survival data. Appendix to An R and S-Plus Companion to Applied Regression.

Giannakou, M.E., Goss, M., Jacobson, J., Vinti, G., Leevers, S.J., and Partridge, L. (2007). Dynamics of the action of dFOXO on adult mortality in Drosophila. Aging Cell *6*, 429-438.

Giannakou, M.E., Goss, M., Junger, M.A., Hafen, E., Leevers, S.J., and Partridge, L. (2004). Long-lived Drosophila with overexpressed dFOXO in adult fat body. Science *305*, 361.

Gronke, S., Clarke, D.-F., Broughton, S., Andrews, T.D., and Partridge, L. (2010). Molecular evolution and functional characterisation of Drosophila insulin-like peptides. PLoS Genet *6*, e1000857.

Ignatiadis, N., Klaus, B., Zaugg, J.B., and Huber, W. (2016). Data-driven hypothesis weighting increases detection power in genome-scale multiple testing. Nature methods *13*, 577-580.

Janky, R., Verfaillie, A., Imrichova, H., Van de Sande, B., Standaert, L., Christiaens, V., Hulselmans, G., Herten, K., Naval Sanchez, M., Potier, D., et al. (2014). iRegulon: from a gene list to a gene regulatory network using large motif and track collections. PLoS computational biology *10*, e1003731.

Slack, C., Giannakou, M.E., Foley, A., Goss, M., and Partridge, L. (2011). dFOXO-independent effects of reduced insulin-like signaling in Drosophila. Aging Cell *10*, 735-748.
